# Childlessness: Concept Analysis

**DOI:** 10.3390/ijerph19031464

**Published:** 2022-01-27

**Authors:** Olga Gouni, Gabija Jarašiūnaitė-Fedosejeva, Burcu Kömürcü Akik, Annaleena Holopainen, Jean Calleja-Agius

**Affiliations:** 1 Cosmoanelixis, 104 37 Athens, Greece; 2 Department of Psychology, Faculty of Social Sciences, Vytautas Magnus University, LT-44248 Kaunas, Lithuania; gabija.jarasiunaite-fedosejeva@vdu.lt; 3 Department of Psychology, Faculty of Languages and History-Geography, Ankara University, Ankara 06100, Turkey; komurcu@ankara.edu.tr; 4 Department of Clinical Child and Family Studies, Faculty of Behavioural and Movement Sciences, Vrije Universiteit Amsterdam, 1081 HV Amsterdam, The Netherlands; a.holopainen@vu.nl; 5 Department of Anatomy, Faculty of Medicine and Surgery, University of Malta, MSD 2080 Msida, Malta; jean.calleja-agius@um.edu.mt

**Keywords:** childlessness, child-free, antinatalism, infertile, barrenness, sterile

## Abstract

The purpose of this concept analysis is to explore childlessness and provide understanding to professionals involved in the field of infertility. Walker and Avant’s method was used to identify descriptions, antecedents, consequences, and empirical referents of the concept. A model with related and contrary cases was developed. The analysis was based on the definition of the term in major dictionaries in the Greek, Lithuanian, Finnish, Maltese, and Turkish languages, while further literature searches utilized the Web of Science, PubMed, PsychInfo, Medline, Google Scholar, and National Thesis Databases. The literature search was limited to papers/books published in the authors’ national languages and English. As a result, childlessness is defined as the absence of children in the life of an individual, and this can be voluntary or involuntary. However, the deeper analysis of the concept may be preceded and amplified through cultural, psychological, biological, philosophical, theological, sociological, anthropological, and linguistic aspects throughout history. These elements presented challenges for childless individuals, ultimately influencing their choices to resort to alternative ways of becoming parents, such as in vitro fertilization (IVF), surrogacy, adoption, or other forms of childbearing. Historically, childlessness has been viewed with negative connotations due to its potential impact on the survival of the human species. This negativity can be directed even to individuals who may decide to opt to voluntarily remain childfree. The long-term impact of the experience, both on an individual and collective level, continues to cause pain to those who are involuntarily childless. In conclusion, health professionals and other stakeholders who have a deep understanding of childlessness, including the antecedents and attributes, can minimize the potential negative consequences of those factors contributing to childlessness, whether voluntary or involuntary. In fact, they can capitalize on a powerful impact of change adaptation by providing support to those in their practice to recover the lost homeostasis.

## 1. Introduction

Childlessness is defined as the absence of children in an individual’s life [1]. Childlessness can be considered involuntary when an individual is unable to have children for medical reasons, whether known or unexplained [2]. According to the World Health Organization [2], “infertility is a disease of the male or female reproductive system defined by the failure to achieve a pregnancy after 12 months or more of regular unprotected sexual intercourse”. Childlessness can be considered voluntary when a human being deliberately chooses not to have children. A “childfree” definition is usually used in this case. Finally, childlessness can be considered determined by circumstances when the person wants to have children, but the first pregnancy is delayed and, in the long run, is left without children for social or physiological reasons [3].

### 1.1. Historical Background

The negative attitudes towards childlessness, especially childless women, have their roots in antiquity. In many cases, those attitudes were supported by existing law. Unfortunately, we can still trace ancient mindsets in the cultures of almost all countries participating in this concept analysis. Maltese figurines of the Goddess of Fertility from the late Neolithic (3000–2500 B.C.) found in hypogeums in Malta, as in other prehistoric cultures, represent the importance of praying to the deities from the dawn of humankind to be liberated from the “curse” of childlessness [4]. In Babylon, Assyria, and Egypt, a husband could divorce a wife if she was infertile. Another example is the famous Law of Hammurabi (1750 B.C.). According to it, an infertile woman can give a slave to her husband to “impregnate” (the use of “impregnate” implies that the male is the predominant gender behind a conception, an attitude that held strongly until very recently, originating from the Vedic traditions according to which, the earth layer of the triparte world was the one (considered the mother) that received the water falling from the Heaven (the invisible layer, the residence of God(s) and considered to be the father) in the form of rain, storm, etc., and then vegetation came out) the child is born, the master’s wife raises the child as if her own and the slave gets her freedom for the service to the master [5].

Many of the women in the Bible are described as being barren. In the Old Testament, Abraham’s wife “Sarah was barren; she had no child”. Sarah encouraged Abraham to have a child with a young woman, Hagar [6]. Rachael asked Jacob to have intercourse with her servant Valla to have a child [6]. Location Uteri (ancient Rome) allowed patricians to have a child through another woman without having to go through the hassle of pregnancy [7]. In Homer [8], Megapenthis was the child of Menelaos and a slave because the Gods did not give Helen a child [8]. In ancient Sparta, other women were allowed to come and live in the house of the husband to maintain the continuity of the family [9].

Resonating the above-mentioned Babylonian, Assyrian, and Egyptian sources, in old Greek legislative documents, if a woman could not have children, the husband was allowed to have sex with another woman [10]. Initially in Mani and then in other areas in Greece (Trikeri of Thessaly, Kerkyra (Corfu) and Crete) and despite the resistance of the Orthodox Christian Church, we find the practice of synkria (syn + kyria, kyra (συν + κυρία/κυρά)) that was present until the end of the 19th century. According to this practice, when a wife could not have male children or no children at all, her husband could get married to a second woman without getting a divorce first, and the two marriages were both valid. The second wife was called synkria *(σύγκρια*) or synkormissa *(συγκόρμισσα*) and replaced the first wife in the child-bearing process so that the socially needed male descendant was born. The relevant agreement was even part of a dowry agreement, to which the first wife and the parents of the second one contributed. In Mani, the position of the second wife (synkria) was under the condition of the birth of a male child, while the housewife (“mistress of the house”) remained the first wife. When the male child was born, the first wife became co-godmother, replacing the biological relation with the spiritual one. However, if the second wife (synkria) gave birth to female children, then her daughters were considered inferior to the daughters of the first wife and inherited only the paternal property. In addition, in case the second wife (synkria) remained childless, she ran the risk of being kicked out of the house as a sinner or, in the best scenario, she would remain in the house as a “servant” (*ψυχοκόρη*) [11].

In the ancient Mesopotamian cuneatic sources, people used infertility to marginalize each other in social life. To prevent the problem of infertility and miscarriage, people prepared drugs using various plants, stones, and minerals, or they resorted to “magic” as a part of the treatment. However, the meaning attributed to the child by the woman and the man was not always positive, in which case the pregnancy was either continued as a conscious choice or otherwise it was terminated, again with the help of herbal drugs [12].

In the late Ottoman advice books, infertility was discussed from medical, moral, and social aspects for both women and men [13]. For example, in his book *Ukm ü Ananet* (Infertility and Impotence), Ömer [14] dealt with infertility as a whole with its physiological, psychological, medical, and social consequences. However, he devoted a significant part of the book to female infertility. He stated that while infertility prevents fertility, impotence can only be an inability to have sexual intercourse (as cited in [13]). In Ottoman, although it is thought that it is much easier to find a cure for impotence, female infertility has been seen as a much more complex and difficult issue to treat [13].

In the history of Turkey on childlessness is the article “The Greatest Problem of Nations: Childlessness” written by Selim Sırrı [15]. In this article, which was written with the main focus on social sensitivity regarding child mortality, the importance of the child was associated with the population issue and it was stated that the biggest problem of the nations was childlessness. Τhe long years of war had dragged the whole of Anatolia and its people into negative conditions, and the importance of increasing the population by preventing child deaths was emphasized in terms of national defense and the future of the country (as cited in [16]). Moreover, male infertility and impotence attracted great attention as a medical problem in the late 19th and early 20th centuries, and many books were published on its treatment [13].

Historically in Lithuania, childlessness was considered a great dishonor. The women in Pagan times prayed to the Goddess of Fertility Zemyna and sacrificed a white hen to the Goddess to get pregnant. The women of Catholic Lithuania prayed to conceive, requested masses for this intention, and made many promises and offerings to God [17].

In the earlier written Lithuanian sources or the notes collected from older people’s memories, it is mentioned that a childless couple always lived in shame and humiliation. It was considered God’s punishment not only on Earth but also after death. According to Kriauza [18], it was thought that a married, childless woman would have to “wander in the dark, soak in water or carry the devil’s children after death”. Infertility was always blamed on the woman in the first place: she was called using humiliating words, mocked, and reproached not only by those around her but also by her husband and mother-in-law. She was insulted by the words such as “childless”, “empty basket”, and accused of being possessed by evil spirits [19].

The historical background in Finland differs partly from the other presented countries, since already in the earliest written Finnish sources that have mentioned the word “childless” or “childlessness”, both negative and positive notions about the phenomenon can be found [20]. It talks, for instance, about childless people being the happiest, but also about someone with a “bad life” being threatened with childlessness. Later on, in the 19–20th centuries, women were almost always blamed for childlessness and families with no or only one child were not appreciated [21]. In fact, only around that time motherhood started to become strongly associated with womanhood ([22], as cited in [23]). Yet, already in the 1980s, the majority of Finnish women did not believe that they needed to have children to fulfill their roles as women [24]. As many as 25% of Finnish women born in the 20th century were, in fact, childless [25]. Accordingly, childlessness was rather common in preindustrial Finland because marriage and children needed to wait until a couple managed to live an independent life [25].

### 1.2. Modern Times

Throughout the 20th and 21st centuries, we can still see the remains of deeply ingrained attitudes. In many societies, the mother figure has a central place as she represents security, continuity, sacrifice, and unconditional love [10,26,27]. Having children seems to be a significant element of social coherence and social organization. Being childless can be a stigma, causing a lot of problems not only to the spouses and their marital relationship but also their social image. Thus, people tried to find alternative ways to evade the negative consequences of being childless from time immemorial. Among these “survival strategies”, there has been adoption, parallel marriage, or resorting to another man or another woman who could help to bear a child.

**Figure 1:**
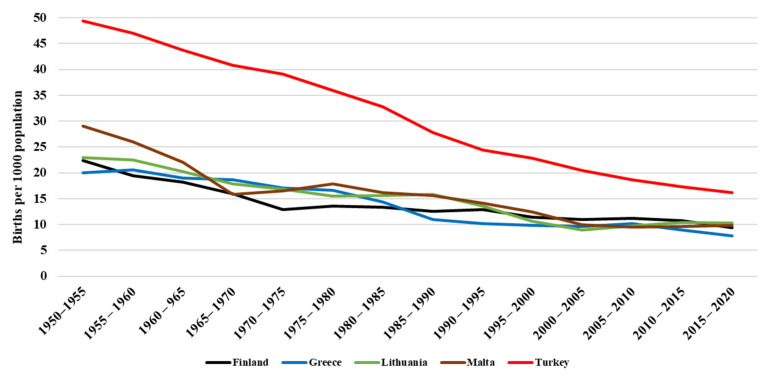
Birth rate by country in 1950–2020 (births per 1000 population). Source: [32].

**Figure 2:**
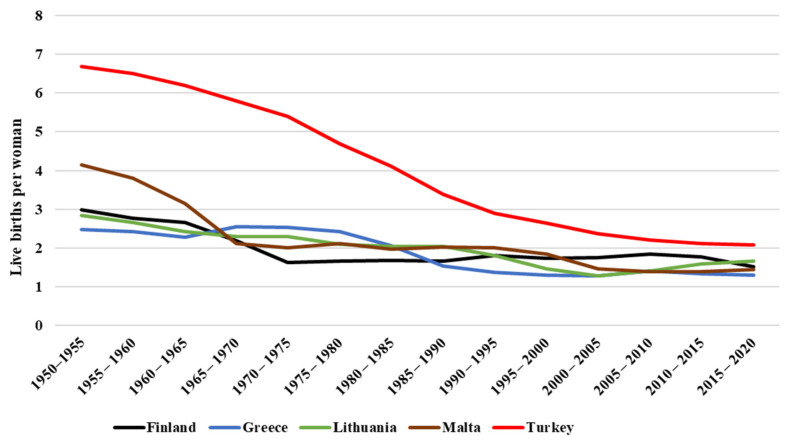
Total fertility 1950–2020 (live births per woman). Source: [32].

In modern times and recent years, as in the rest of the world, it is observed that birth rates have been decreasing in Greece, Turkey, Malta, Finland, and Lithuania (Figure 1) as well as live births per woman (Figure 2) [28,29]. Due to the technological advances in biotechnology and genetics, many couples who have conception challenges attempt to undergo in vitro fertilization (IVF), surrogacy, or egg/sperm donation. In Lithuania, Finland, Turkey, and Malta, surrogacy is not legal, but IVF, adoption, and artificial insemination procedures are regulated by law [30,31]. Greek legislation allows surrogacy, adoption, or IVF. Due to technology interventions, there has been a triple categorization of mother: the genetic mother (the ovum is hers), the birth or gestational mother who gestates and gives birth to the baby, and the social mother who raises the child [10].

Besides considering the world population rates, it can also be noticed that the numbers of those who prefer childlessness are increasing, and they have become more visible [33]. It seems that childlessness in modern society is becoming a particularly favorable status—it is easier for an individual without family responsibilities to participate in career and material wellbeing [34]. According to Sobotka [35], the *ultimate market society is childless.* However, being childless in different countries has consequences, whether voluntary or involuntary. In Turkey, being a childless woman is a “disturbing” issue for the general public because having children is still an important and generally accepted norm [36]. This norm is valid for the other countries included in this study (e.g., [10,37]). As can be seen in the paragraphs to come, Finland differs in some aspects from the other included countries regarding the attributes and consequences of childlessness (e.g., [25]).

### 1.3. Aims and Goals of the Concept Analysis

The present concept analysis aims to explore the selected concept of childlessness in the five selected languages of the researchers (Greek, Lithuanian, Finnish, Turkish, and Maltese). Its purpose is to clarify the meaning, identify the defining attributes through discovering existing variables within the relevant national languages and cultures, and spot similarities or possible differences within them. The aim is to acquire a better understanding of the phenomenon, highlighting the consistency and coherence of the term connected with the existing body of knowledge, provide evidence by clarifying any ambiguity, and distinguish between the normal, ordinary, and scientific language usage of the concept so that an operational definition can be presented. It also aims to better describe the inner and outer forces at work behind voluntary or involuntary childlessness, explain unclear aspects, and help understand various forms of reasoning such as child-bearing decision making and finally give meaning to human experience [38]. The current paper can provide a better understanding of the phenomenon to professionals involved in the field of infertility. With such a better understanding, they may provide better care for childless people, whether it is voluntary or involuntary. Yet, the information included in this paper can offer insights for anybody, who is, or who knows someone who is childless.

## 2. Methods

**Figure 3:**
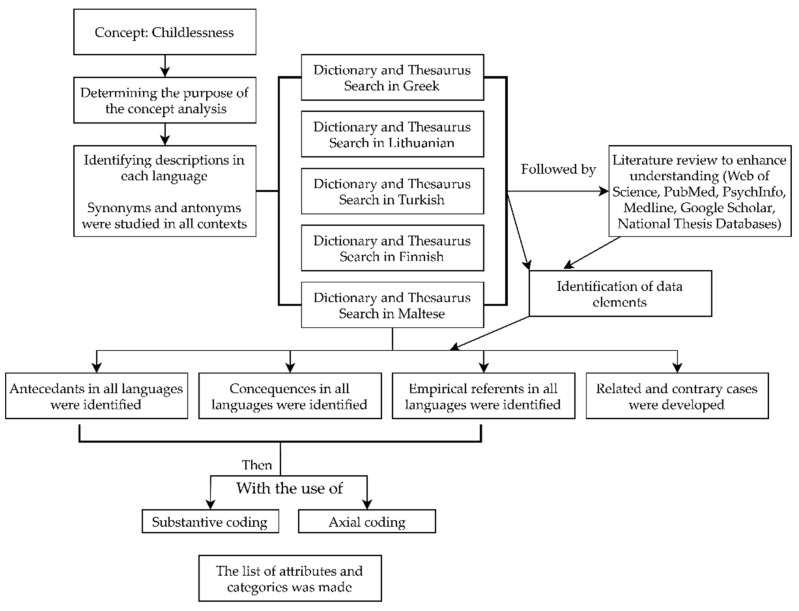
The stages of childlessness concept analysis (based on Walker and Avant’s model).

Dictionary and thesaurus searches were made in Greek, Lithuanian, Finnish, Turkish, and Maltese, followed by a literature review with the aim of spotting the emerging categories and enhancing data collection and analysis to better understand the concept. Definitions from various fields were included. Then, elements related to the true meaning of the concept were found and were assessed in terms of relevance, completeness, and amount of information attributed to critical thinking [39,40]. The test of sufficiency and necessity were observed throughout and the attributes of the concept were checked to make sure they did not apply to dissimilar concepts [39]. Substantive codes were used to accommodate the fit, work, and grab principle. Axial coding was used to define categories. Different cases were then explored to crystallize the demarcation of the concept. Attributes were compared with other emerging categories to check whether the relationships still held. The emerging categories were studied to add understanding so that the phenomenon could be wholly understood [41]. Information was added to the sections only for those countries where information based on scientific sources was found. The flow chart showing the stages of concept analysis followed in this study was given in Figure 3.

## 3. Results

**Figure 4:**
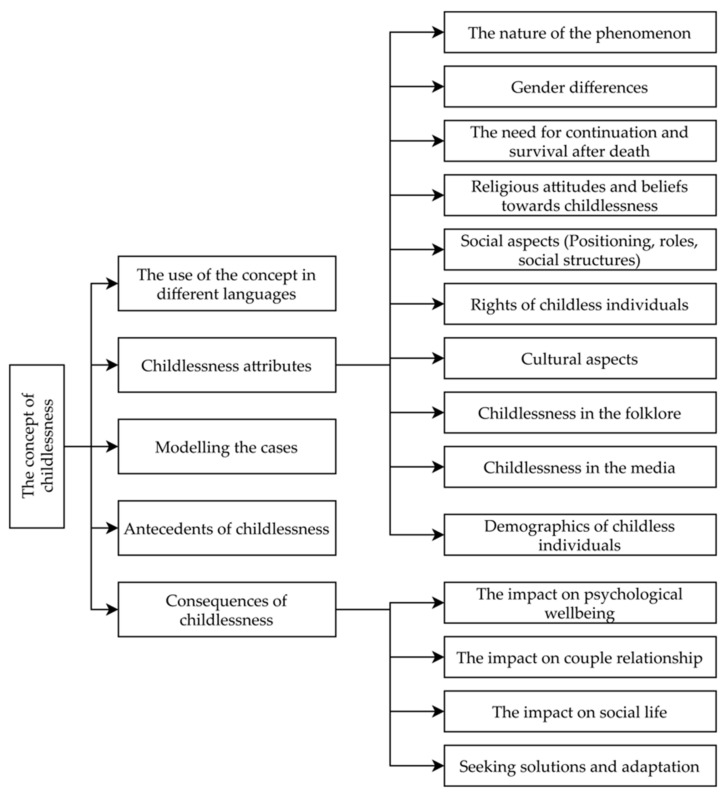
A conceptual map of the results of childlessness concept analysis.

The conceptual map of the results of childlessness concept analysis, based on Walker and Avant’s model, is presented in Figure 4. First of all, we present the use of the concept in different languages (Greek, Lithuanian, Finnish, Turkish, and Maltese). Secondly, the attributes based on literature analysis and emerging themes are analyzed. Third, we present the cases (a model case, a borderline case, a related case, and a contrary case). Finally, antecedents of childlessness and consequences that also emerged from literature analysis are presented.

### 3.1. The Use of the Concept

Childlessness, which may be by choice or circumstance, is distinguished from antinatalism, a philosophical position and a social movement that supports that having children is not ethical, thus having no children is promoted. In this paper, the focus will be on childlessness as a general concept. The defining attributes of childlessness were determined by: (1) the study of the dictionary definitions of the concept in the specific languages (Greek, Finnish, Lithuanian, Maltese, and Turkish); (2) the dictionary studies of relevant concepts in the same languages. Generally, the English version of the word is given and its equivalent in the relevant language follows in parentheses. However, sometimes in other languages, it was not easy to find an English word that describes the same term. In these cases, the national term comes first followed by the explanation in English.

#### 3.1.1. Greece

The dictionary of Babiniotis [42] defines childlessness (*ατεκνία)* as the state of not having children. The word comes from the prefix *a-* expressing negation or absence and the root *τικ-* as found in the ancient Greek word *τί-τκ-ω* (with present duplication and inversion of consonants) (transitive verb) used for women, not for men (the word used for men was *γεννώ*), to mean *bring to the world.* The root *τικ* comes from the Indo-European tek- which means *give birth to*, but also *witness* and the Sanskrit word *tâk-man-* which means “child, descendant”. The root is also referred to relate to the ancient German root *degan* and the ancient Scandinavian root *pegn* or ancient English *peg(e)n*, which means “*warrior*, *servant*, *young man*”. The same root *τικ-* is found in child (*τέκ-νον*), when talking about *the stage in which smth manifests, comes to the world*, *labor* (*τόκ-ος*, *τοκ-ετός*). It is interesting here to also see that the root τοκ- as in τόκος which is also used to describe labor is the same word used to show *more* or *profit* or the *value that a person pays to have what this person wishes to make use of*. The word *γεννώ* (give birth) which is the word used for men originally comes from the root *γεν-* which is also met in words like genre, nation (*γέν-ος*), *brave* (*γενεα-ιος*>*γενναίος*) and *birth*, *labor* (*γέννα*>*γεννώ*) (of which we see the word *γονιμοποιώ*). The man is the one *who makes it possible for a woman to bring a child to the world by providing the information for the new organism* (the child) *who will continue the nation, the family, the genre* (*γένος*) *by being the brave one who will fight for it*, the nation. It is also important to see that the root *γεν-* also describes *good understanding*, *knowing a person very well* as in the sentence *σ’ έχω γεννήσει εγώ* = I have given birth to you which is associated with the male side, not the female. Moreover, the meaning of *γεννώ* as “*mentally conceive*”, “*mentally create*”, and “*materialize* in the meaning of bringing something new to the world” as found in sentences such as “*γεννώ ιδέες* = bring ideas to the world” or is used to describe “*the one who has the innate skill of something*” as in the example “*γεννήθηκα έξυπνος* = Ι was born intelligent”: these words have been used so widely in science for example but they are associated with the male quality in the Greek language and culture in the past, still existing in some areas in the mentality of the people. The word childbirth (*γέννα*) is the word widely used today in Greek for childbirth in Greece and it comes from a root initially kept for men, while to describe the act of labor the word (*τοκετός*) is used and to describe the woman who is to labor the word (*επίτοκος*) is used.

Further research of the concepts close to childlessness revealed a number of related words. The translation beside the Greek words is only part of the meaning such as “*ασπερμία* = the state when no sperm is produced, no seeds are present”, “*ακαρπία* = the state when a plant does not give any nuts or fruits”, “*ακληρία* = the state when a person has no heirs and thus cannot be inherited” (all words have the most interesting content which is presented elsewhere in this paper or in other publications) and the adjectives “*άπαις* = without children”, “*στείρος* = not giving any products, but also the one not polluted by other elements”, “*άγονος* = inability to reproduce”, and “*ατέκμαρτος* = the one that cannot be concluded, unknown”, which were mainly used in fields such as agriculture, law, philosophy, economics, zoology, and sociology.

Finally, a study of the main antonyms of the concept can better clarify the understanding of childlessness. The term that describes the state of having many children (*πολυτεκνία* polýteknia) is the main antonym. However, the words parenthood (γονεϊκότητα), and more specifically motherhood (*μητρότητα*) or fatherhood (*πατρότητα*) and words such as fertility (*γονιμότητα*) and the Greek words *καρπερή* (producing too many seeds, nuts, or fruits), *κουνέλα* (female rabbit), *αγορομάνα* (only giving birth to males), and *κοριτσομάνα* (only giving birth to females), are rich in meaning. To start with, the word parenthood (*γονεϊκότητα*) which is a term that describes both mother or father, comes from the same root gon-, of the Indo-European gen-, which has already been mentioned above. It is interesting that the word that is associated only with the male has been used for females as well. The understanding of the terms of parent (*γονεϊκότητα* and *γονεύς/γονέας*) allows us to conclude that in the case of childlessness, there is a break in the continuity of the ancestry and perhaps a major threat for the survival of the people. Connected to the root *γεν-* in the meaning of knowledge and understanding is the role of the parent to be wise enough to guide the child in their life and make wise decisions concerning the child. The state of childlessness implies that, first of all the lineage stops, the whole human species is in danger, and wise leadership does not apply.

In close connection with parent/parenthood (*γονεύς*/*γονεϊκότητα*) is the word fertility (*γονιμότης* “gonimótēs”/*γονιμότητα* “gonimótita”), also an antonym. The word comes from the same root *γον- γεν*-. It is the state of being able to have children (humans, also animals) or deliver products, seeds, and fruit, but also able to deliver outcomes such as artistic, mental, or literary works. Thus, it is the state of supporting creativity and productivity on a material level but also social, philosophical, scientific, cultural, and artistic level. It implies strong imagination that connects the visible with the invisible, acceptance that gets what is conceived and nurtures the ideas conceived so that they can develop in a supportive environment, and creative powers to bring the conceptions of the mind (through imagination) to birth and emerge on the physical level. Thus, childlessness lacks such qualities or, if present, these are imperfect, explaining why childlessness is associated with imperfection, impotence, and why it bears such humiliating connotations.

The Greek word for motherhood (*μητρότητα*) describes the state of being a mother (*μητέρα, μήτηρ*). The word μήτηρ (proto Hellenic word *mā́tēr* associated with the proto Indo European *méh₂tēr,* from which we have the modern word “*μητέρα*” but also the words *μάνα, μαμά*) is the one from whom everything and everybody owes their existence, the cause that leads to the evolutionary emergence of all else. Its association with the Greek word *μήτρα* (=womb) is obvious. Thus, we could say that biologically the woman who gives birth to a child being gestated in her womb is the mother but beyond that μητέρα or μήτρα is the space, the environment in which values, idea(l)s, civilization and the rest gestate before they are born and radiate into the world. Going even further, we could also see traits of science related to the creation of the universe as it may imply the process of the whole universe coming out of the same “primal bubble” according to the theory of the exponential expansion of space in the early state just after singularity [43]. Thus, childlessness is the state that gestates nothing of value, does not participate in any evolutionary process; it is a state of imperfection, not serving any good cause and deserves little if any respect. It is also very interesting to see that the word stepmother (*μητρυιά/μητριά*) bears a very negative meaning, despite the fact that it comes from the same root *μη-* of *μήτηρ* (mother) as it implies cruelty and absence of any nurturing care to the child. Furthermore, the words *αγορομάνα* (the mother who gives birth only to male children) and *κοριτσομάνα* (the mother who gives birth to only female children) are often used to place a special emphasis on the kind of woman one is. The male birthing mother is highly appreciated while the one who gives birth to females is of a lower social status. Finally, the word *κουνέλα* (female rabbit) is a vulgar word used to describe a woman who gets pregnant too easily and gives birth to many children.

The Greek word for fatherhood (*πατρότητα*) comes from the proto Indo-European root “*ph₂tḗr*”. Of which the Latin pater, the Sanskrit “पितृ” (pitṛ), the ancient Armenian “*հայր*” (hayr), and the protogermanic “*fadēr”* (ancient English fæder > English father, German *Vater*) originate. The word describes the genitor, the source of origin, the one who creates, initiates, introduces, discovers, or builds, and generally the one who acts in the name of God. Thus, a father (*πατήρ*) is the wise person, the leader, the inspirator, the head and the decision maker of the family. He is a person of honor, which is why it is used to refer to the body of clergy (God’s representatives on Earth). It is also a term used to describe the significant ones who established philosophies, theories, and present literary, scientific, or academic works. The root is also found in the word motherland (*πατρίς*/*πατρίδα*) which although in English comes from the word mother, in almost all other languages comes from father. However, although we can very well understand why a motherland (*πατρίς*) is associated with father *(πατήρ*), in Greek, there is a very special aspect as the word root is *πατήρ* but the gender is female, thus the same word combines the two aspects male and female in one. The term stepfather (*πατρυιός*/*πατριός*) used to describe the husband of the mother of a child but not the biological father of this child, also comes from the same root and as in the case of the female equivalent, it usually carries a more negative meaning, perhaps less than in the case of the stepmother, nevertheless implying lack of wise leadership and respectful child upbringing. In general, a male who is not a father because he is childless lacks all the above-mentioned qualities, does not initiate anything, is not a creator and male childlessness is a state of not giving out anything of value in either the physical dimension (e.g., children, warriors) or the mental (e.g., theories, philosophies, works of meaning), and does not participate in land protection. Certainly, male childlessness is not a state of honor. All the above words shed light on the better understanding of the deepest meanings and can explain the psychological, social, or other burdens of the concept throughout time, as will also be discussed in other parts below.

#### 3.1.2. Turkey

There is no clear information about when the word childless (*çocuksuz*) or childlessness (*çocuksuzluk*) was first used because there is also an unwritten oral tradition period in Turkish literature. However, the subject of childlessness takes place in epics and tales [44,45]. According to the oldest source that is available, the word child (*çocuk*) was mentioned for the first time in a work published in the 19th century [46]. The meanings of the word child (*çocuk*), boy or girl of a young age, means that they are in the developmental period between infancy and adolescence, and metaphorically, the one who is less old among the elders, a person who behaves in a way not appropriate for the elders, but rather a characteristic of younger, immature people, and lastly, someone who does not have sufficient experience and ability in a particular field. As can be seen from the definitions, the last two meanings are negative. The word child (*çocuk*) is the root of childlessness (*çocuksuzluk*) in Turkish.

The Turkish Dictionary of the Turkish Language Association [47] (the revised version of the 11th edition of the Turkish Dictionary published in 2011 by the Turkish Language Association, which has been published since 1945, and presented on the public network; the Turkish Dictionary is constantly updated depending on the developments in the Turkish language) defines childless (*çocuksuz*) as somebody without a child. The word childless (*çocuksuz*) is a word formed by adding a negative suffix expressing absence, lack of things to the word child (*çocuk*). The synonym of childless (*çocuksuz*) is the word infertile/barren (*kısır*). It is defined as unable to reproduce, not giving offspring (human or animal), does not produce (soil), and that in which no reproductive event occurs (living cell, nucleus, etc.), and sterile. Metaphorically infertile/barren (*kısır*) means inefficient, useless, fruitless. From this point of view, the word infertile/barren is a word that has negative meanings and can imply pity, embarrassment, and inadequacy towards the person who is mentioned as infertile. While the word childless (*çocuksuz*) has a more positive/neutral meaning, the negative meaning is more dominant when the word infertile/barren/sterile (*kısır*) is used to refer to someone. In addition, the word infertile (*infertil*) is not found in the Turkish Dictionary because it is not Turkish, but a word loan from English that has “settled” in Turkish and is frequently used especially in academic literature.

The word childlessness (*çocuksuzluk*) is a word formed by adding two consecutive suffixes (*-suz* and *-luk*) to the word child (*çocuk*) and defined as the condition or state of being childless. The synonym of childlessness is the word infertility/barrenness (*kısırlık*) which is defined as the status of being infertile and metaphorically as inefficiency. While the word childlessness (*çocuksuzluk*) has a more positive/neutral meaning, the negative meaning is more dominant when the word infertility/barrenness/sterility (*kısırlık*) is used to describe the situation. Infertility (*kısırlık*) is perceived as a problem that needs to be solved, and/or something negative that happens to the person. As mentioned above, the word infertility (*infertilite*) is a word loan from English.

Another term is “voluntarily childless” (*gönüllü çocuksuzluk*) or “childless by choice” (*tercihen çocuksuzluk*) defined as not having children based on one’s own preferences, not due to circumstances. Although this is not in the current Turkish dictionary published by the Turkish Language Association, it is often seen in the articles written from a feminist perspective. Finally, the word childless and childfree are translated into Turkish as the same (*çocuksuz*), but the difference in meaning is known and academics use them to refer to voluntary childlessness. There is an increase in voluntarily childless people numbers in Turkey and despite the criticism, the concept itself has a positive meaning.

Trying to understand the concept of childlessness, it is necessary to consider the antonyms of the word. The word parenthood (*ebeveynlik*), which is formed with a constructional suffix that comes to the word parent, means being a parent and having the responsibilities of the parent(s). In Turkish, motherhood (*annelik*) has two meanings, (i) the quality or state of being a mother, maternity and (ii) maternal behavior; while fatherhood (*babalık*) is defined as the state of being a father.

When the antonyms of the word childless are examined, we encounter the word “having a child/with child” (*çocuklu*), which is formed by adding the suffix (*-lu*) to the word child (*çocuk*), which means a person who has a child. Another word, parent (*ebeveyn*), has passed from Arabic to Turkish; it is a plural form that is used for both mother and father. The word mother (*anne* or *ana*) has two meanings, (i) woman with a child, and (ii) female animal with offspring. Father (*baba*) has several meanings, male with child, the man who contributed to the birth of the child, someone who provides benefit to a country or a community, and metaphorically an understanding, good-natured male, the head of a gang that does dirty and clandestine business such as arms trafficking, money laundering and drug dealing, protector, someone full of paternal feelings, and ancestor. In addition, a woman may breastfeed another woman’s child in case the biological mother has breastfeeding challenges and/or has no milk to nourish the newborn, or is sick/unable to breastfeed. In this case, she is called a “wet nurse” (*sütanne*). Although this practice is not very common today, it can still be seen in some parts of Turkey [48].

Other antonyms are stepmother (*üvey anne*/*analık*/*cici anne*) and stepfather (*üvey baba*/*babalık*/*cici baba*), which means mother and father who are not biologically the parent of the child and mother and father who mistreat their child. In Turkish, the term stepmother has a negative meaning as in many other countries and is introduced in Turkish literature as cruel, tormenting, treacherous characters, and a similar perception exists for stepfathers. The meaning of another antonym foster family (*koruyucu aile*) is a voluntary family that undertakes all kinds of care and responsibilities of an orphan or child in need of care within a certain period of time according to the relevant law. In Turkey, there is a separate concept of a person having legal custody of a child, its boundaries are crossed by adoption law. Finally, fertility, which is another antonym of the concept of childlessness, is defined as the state of giving birth, perhaps to more than one child, being fertile, and has a positive meaning. Similarly, as in Greek described above, in Turkish the phrase “like rabbits” (*tavşan gibi*) is used to describe a couple who have an intensive sexual life and many children in slang language.

#### 3.1.3. Lithuania

Lithuanian dictionary [49] defines childlessness (*bevaikystė*) as the state of having no children. The word comes from the prefix “*be-*”, expressing absence, lack of things, and the root “*vaik*”, which is the root for all the words related to children. The same word is used when describing voluntary, involuntary, or circumstances determined childlessness. It is also interesting that without the prefix “*be*” (in English “without”), the word means childhood. The Lithuanian dictionary gives the reference to the notes of Jurgis Pabrėža (1771–1849) who was a priest, Franciscan, doctor, botanist, and one of the most prominent educators in the 19th century and to the newspaper in 1863 “Keleiwis isz Karalaucziaus Broliams Lietuvininkams Žines pranesząs”, 7p., editor: preacher Fr. Kurschat [50]. It can be assumed that it is the first document that the word childlessness was mentioned in writing.

The adjective childless (*bevaikis*, *-ė*) describes a person who has no children. The word comes from the German–Lithuanian dictionary by Friedrich Kurschat in 1870 (Volume 1) and 1874 (Volume 2) [50]. So, it can be concluded that the earliest we could find childlessness and childless mentioned in Lithuanian is the 19th century, even though the concept was used a lot earlier in folklore and speaking language.

The synonym of childlessness is infertile (*nevaisingas*) meaning the one who cannot have children. The noun is infertility (*nevaisingumas*). Other synonyms are sterile/sterility (*sterilus*/*sterilumas*) meaning infertile, and also the one free of germs. This word does not have such a positive meaning when referring to a couple who cannot have children, but on the other hand, it can have a positive meaning as well. The third antonym is impotence (*impotencija*), meaning inability in general or sexual disability of a man.

The word parenthood (*tėvystė*) has two meanings: being a parent and bearing the responsibilities of the parent(s). Lithuanian language also has separate words for fatherhood and motherhood. Fatherhood (*tėvystė*) means being a father, the duties of the father (parents). This concept is also used when talking about a group of people originating from one father, family, nation, etc. It could be assumed that without being a father, one does not belong to a group or nation. Motherhood (*motinystė*) has three meanings. The concept describes the condition of a woman during pregnancy, childbirth, and breastfeeding; being a mother; mother’s position, duty. Another meaning is the mother’s feelings for her children and the mother’s biological relationship with her children.

The concept of a father (*tėtis*, *tėtė*, *tėvas*) means a man to his children. Lithuanian “*tėvas*” is also used when describing a woman’s spouse, man, as well as an elderly man (when you approach a person). By religious people, “*tėvas*” is also used when describing the patriarch, creator. This concept is also used for the surname of a priest, clergyman, or monk and as the proverb of God. So, being a father has a meaning related to having children, but it is also used when talking about God, creator, and the father of the Christian church. Not only can God create, but the man himself can be a creator as he supports the continuity of humanity. A concept of mother (*mama, motina, mamytė, mamužė, mamutė, mamulė, mamulytė, mamalė, mamaitė*) means a woman as perceived by her children. From the many different words used to describe a mother, we could see the importance of this concept historically. According to Balys [51], there are more than one hundred familiar words for mother. They all contain the same root, “*mot*”, “*ma*” (Sanscrit—*matha*, Lithuanian—*motina*, *mama*), to which many suffixes are added to express tenderness and affection (e.g., *motušė*, *motužė*, *mamužė*, *mamulytė*, etc.) [17]. Lithuanian “*motina*” is also used for a wife, but Lithuanian “*žmona*” is more common than “*motina*”. A concept of “*motina*” is also used when describing a female animal having offspring. Besides, the concept “*motina*” is used when talking about the only female in the bee family that can lay eggs, the queen bee, and old potato from which new ones grow, and the beginning, the source, the basis of something.

The word parents (*tėvai*), means a father and a mother. Lithuanian “*tėvai*” is also used for ancestors: father/mother of parents. Moreover, for people respected by all: second parents by the spirit: priests, teachers, etc. You could earn respect by being a parent. The English “parents” in Lithuanian also has the meaning of biological parents (*gimdytojai*). Other antonyms are stepfather (*patėvis*), stepmother (*pamotė*), and foster family (*globėjai*). Stepfather is not the biological father, but a mothers’ second husband to the first man’s children, or husband to his wife’s children from her previous marriage. Stepmother means not the biological mother, father’s wife to children from previous marriages, or even less privileged wife. In folklore, Lithuanian “*pamotė”* has a very negative meaning and, in fairytales, is often depicted as a witch. In tales, a stepmother is seen as jealous, ill-tempered, and mistreating her stepdaughter. Foster family means the one who takes care, cares, and helps, also entrusted by law with the custody of others; a court-appointed guardian. In Lithuania, a person with legal custody of a child has a separate concept—“*vaiko globėjas*”.

The word fertility (*vaisingumas*), meaning the quality of being fertile. The adjectives “fertile, fruitful” (*vaisingas*) meaning not only able to have children, breed well, healthy but also having or bearing much fruit, suitable for a good harvest, yielding a good harvest, fertilizers, yields; characterized by a good harvest; fertilized and giving good results, productive. The last of found antonyms is Lithuanian “*daugiavaikis*”, meaning having many children. The word “*daugiavaikis*” in spoken language has mixed attributes. The positive is respect from growing that many children, but also the negative—poorer living conditions, sometimes unemployed or even asocial parents living on child money.

#### 3.1.4. Finland

The word childless (*lapseton*) appeared in the Finnish language already in the 16th century, while the word childlessness (*lapsettomuus*) was used for the first time in the 18th century [20]. These words are rooted in the word child (*lapsi*) that appeared for the first time in the Finnish language in the 16th century [20]. The origin of the word is unclear, although it has its equivalent in all relative languages, such as the Estonian “*laps*” [52]. The word child (*lapsi*) refers, for instance, to a human being from birth until puberty, to a son or daughter in relation to his/her parents, and to a person growing in a specific environment [53].

The word childless (*lapseton*) is formed by using the suffix -ton, which refers to a lack of something, in this case, a lack of children. As noted by Hallamaa [54], although “lack of something” sounds rather negative, the suffix *-ton* can have both a positive and a negative connotation such as in “lack of worries” (*huoleton*) and “lack of happiness” (*onneton*). Thus, the term childless (*lapseton*) does not in and of itself have either a positive or a negative connotation in the Finnish language.

The dictionary of the Institute for the languages of Finland gives only examples for the words childless (*lapseton*) and childlessness (*lapsettomuus*), but no definitions [53]. These examples are “childless marriage” (*lapseton avioliitto*) and “couples suffering from involuntary childlessness” (*tahattomasta lapsettomuudesta kärsivät pariskunnat*). However, elsewhere these terms have been defined as follows: the adjective childless (*lapseton*) refers to someone who does not have children (voluntarily or involuntarily) or to something where children do not belong [55]. The noun childlessness (*lapsettomuus*) on the other hand, refers to the inability to have children [56]. When childlessness is defined this way, it does not cover the different types of childlessness (e.g., voluntary, involuntary, circumstances determined). As noted by Heinämäki [57], in Finnish there are two adjectives that refer to involuntary childlessness: one that is somewhat more neutral and refers to something that one has not chosen nor planned (*tahaton*), and another one that refers to something that one did not want to happen or something that was against his/her will (*vastentahtoinen*).

Further research of the concepts close to childlessness revealed a number of related words such as an infertile (*lisääntymiskyvytön*) that refers to an individual who cannot have children; barren (*maho*) that refers to being infertile and is used especially when talking about cows; sterile (*steriili*) that refers to being infertile, clean (i.e., without bacteria), or impersonal/unimaginative (figurative, e.g., interior decoration or atmosphere); impotence (*impotenssi*) that refers to an inability in a man to achieve an erection or orgasm; fertile (*hedelmällinen*) that refers to a fertile soil or individual (e.g., a plan or woman), or to productivity (figurative, e.g., thinking or action); fertility (*hedelmällisyys*) that is used when, for instance, talking about soil or a human being (both women and men); and reproductive (*lisääntymiskykyinen*) that refers to being fertile and is used, for instance, when talking about insects (Kielitoimiston Sanakirja, 2020). It is noteworthy that for many of these terms, the Finnish counterparts have either a rather objectifying connotation (e.g., infertile, fertile) or they are used only when talking about animals or nature (e.g., barren, sterile, reproductive), and not about human beings. In fact, only the terms “impotence” and “fertility” are regularly used when talking about people.

When investigating the Finnish antonyms, the search resulted in the following eight terms: (1) parenthood (*vanhemmuus*) that refers to motherhood or fatherhood, but also to older age; (2) parent (*vanhempi*) that refers to a mother or father in relation to their children; (3) motherhood (*äitiys*) that refers to being a mother, becoming a mother, and the relationship that a mother has to her child(ren); (4) fatherhood (*isyys*) that refers to being a father and the kinship that a father has to his child(ren); (5) mother (*äiti*) that refers to a woman in relation to her child or children, animal mother (used by children), some women in high national positions, respectful name for a nun, or the one giving birth (figurative); (6) father (*isä*) that refers to a man in relation to his children, ancestors or earlier generations, some men in high national positions, priests (especially catholic or orthodox), god, inventor or creator, male animal in relation to his offspring; (7) stepmother (*äitipuoli*) that refers to a new wife of the father, and (8) stepfather (*isäpuoli*) that refers to a new husband of the mother [53].

The definitions of the antonyms can offer some important information on the connotations that these terms have in the Finnish language. For instance, as can be seen in the definitions of the terms motherhood and fatherhood, motherhood refers to the relationship (which may be psychological, biological, social, etc.) between a mother and her child(ren), while fatherhood is defined as the kinship (i.e., purely biological relationship) between a father and his child(ren). The same phenomenon can be seen in the definitions of mother and father because among others, the term father refers to “a male animal in relation to his offspring” (i.e., purely biological relationship), while the definition of the term mother does not include the same counterpart. Therefore, one may conclude that fatherhood may potentially be seen as a slightly narrower phenomenon (i.e., only biological) than motherhood. Finally, as in many other languages, also in Finnish the last two words, namely stepmother and stepfather, have a rather negative connotation (i.e., “mean stepmother”). Therefore, in the modern language one may find some newer and more positive equivalents for these terms (e.g., bonus mother, *bonusäiti*).

#### 3.1.5. Malta

Maltese is unique because it is the only Semitic language which is transcribed using an alphabet which is based on Latin script. On the 21 September 1964, Malta became independent and Maltese was proclaimed the national language. The Maltese language is one of the oldest surviving languages dating back from the 9th century and simultaneously one of the newer languages formalized by an alphabet, spelling, and grammar since 1929. The first relatively complete dictionary, Lexicon Melitense-Latino-Italum (Dictionary Maltese–Latin–Italian) had 18,000 Maltese vocabulary words and was published in 1796 by Mikiel Anton Vassalli [58]. There is no mention of fertility or childlessness in this dictionary. However, the term used among the common people (known as “*lingwa tal-kcina*” or kitchen language, referring to the inferiority attributed by the colonials to people who spoke the native language) was “*mielah*” meaning “salted”, suggesting that the individual has too much salt, and is therefore not able to have children. Interestingly, the term “*immellah*” meaning literally “salted” refers to someone who is actually sick or not feeling well. Nowadays, the commonest term for fertility in Maltese is “*fertilita*”, but another less commonly used term is “*tghallil*” according to modern dictionaries. There is no specific Maltese term for being infertile or childless, except the borrowed word “*infertili*” or “*bla tfal*”, meaning without children. The word “*tfal*” which means children, has the same roots as “tafal” which interestingly means clay, which can be molded to make pottery.

Parenthood (“*trobbija*”) derives from “*tarbija*”, meaning baby, so parenthood is taking care of the baby. The word “tqala” means pregnancy, so a woman who is pregnant is called “*tqila*” which literally also means heavy. A mother is called “*omm*” and father is “*missier*”, even though common day language makes use of the words “*mama”* and “*papa*”. The parents are called “*genituri*”, which is derived from the Italian word “*genitori*”. There is a lot of overlap in terms of underlying meaning with the other languages mentioned above, in terms of fatherhood and motherhood. Similarly, as in Greek and Turkish described above, in Maltese the phrase “like rabbits” (“*qishom fniek*”) literally meaning “like rabbits” is used to describe a couple who have many children.

As seen from the above, in all languages, childlessness carries the same or close to the same negative connotations. There are references to the very beginnings of the human efforts to understand the world and the relationships of this visible world with the invisible world, and thus the space of god(s) and the primal agony to secure continuity of life on Earth. The religious/theologian links are evident while the social consequences are still seen today. To further explore the concept, later on, in this paper, there is a review of the literature in each language that was made to cast light on the actual experience of the concept in life and especially in the fields of psychology, sociology, anthropology, history, information and media, literature or folklore studies, as well as gender studies.

### 3.2. Defining Attributes

The definition of the attributes for childlessness were led to the grouping of the attributes that appear which were clustered to allow the broadest insight into the concept. The literature review served to confirm these attributes and offer a better understanding as to what the situation seems to be like today.

Critical attributes are the following:

1 Inability to give birth to a live child. The person cannot give birth to a live child despite the fact that they can conceive or gestate a child or even give birth to a child born dead. Or can give birth only to female children.
2 The person is of reproductive age and has no other barriers.

The main specific attributes of the concept can be classified as below.

#### 3.2.1. The Nature of the Phenomenon

Previous research has offered various perspectives on the nature of childlessness. More specifically, childlessness can be seen as a developmental or traumatic crisis that can have a severe impact on one’s mental health [59,60]. Alternatively, childlessness may be regarded as a multiphase process that is different for each individual [60,61]. The experiential process of involuntary childlessness has been described, for instance, as a journey, roller coaster, and rainbow [62]. The term childfree began to be used in the 1970s among academics and voluntary childless individuals, and today the terms voluntarily childless, childfree, and childless by choice are commonly used to imply that a person has made a conscious choice not to have children [36]. Although there are minor nuances between these terms, all are generally interchangeable [63]. However, the terms infertil(ity), childlessness, and involuntary childlessness are a set of meaning-laden and interchangeable terms regarding the understanding of women’s roles in society and motherhood [36,64].

Up to the mid-20th century, the existing social norms and religious doctrines were the main forces that exerted influence on the generations to have children. Since then, the declining demographics (Figure 1), as well as the advances in birth control, led to the discussion about childlessness as a postmodern phenomenon [65,66,67]. Accordingly, historical demography tells us that in many European regions in the 19th and early 20th centuries, 20% or more of women remained childless. Young adults left the parental household to work as servants and maids and, during that time, they were obliged to stay single and childless [68]. In this way, childlessness was an integral part of the “Western European marriage pattern” [69]. The same phenomenon was observed in the North American family system in which “the single adult was a significant part of the American population” [70]. In pre-industrial times, there were high childlessness statistics. One may wonder whether this is related to the needs of the time and one can dare compare it with the trends today in which being childless is the new trend as it is associated with better career prospects for example. In the cohort studies of the early 20th century, it was commonly argued that childlessness was related to the post-Depression of the 1920s social and economic upheavals [71] and then later with the post-war heavily distorted gender ratios [72]. According to Sobotka [73], the highest levels of childlessness are observed in the cohorts born around 1900 while those born in the 1940s presented the lowest levels. The significant increase in childlessness among young generations and their social consequences of childlessness, the need for the social embeddedness of elderly people and the increasing demands for public social care and health services, and the intergenerational transfers in later life have been studied. Today, childlessness is seen as “a life course process across a series of decision and bifurcation points” [74].

This paper goes beyond the surface of the phenomenon as described so far, and examines the underlying forces which can explain the eons of trauma and pain associated with childlessness. Understanding the underlying forces of a phenomenon can lead to new more effective solutions.

#### 3.2.2. Gender Differences

Women have long been considered the culprits of infertility, and it was thought that if a man could have sexual intercourse properly, he would have to conceive [75,76]. In Greek, as the word *ατεκνία* shows, it is the woman who cannot bring a child to the world. Moreover, not only a child but beyond that, a woman is unable to materialize or manifest as she fails to complete the last stage of actually delivering a living organism. The important thing here is the completion of the (female) task. She may conceive a child or be pregnant but if the child is not born (alive), then she is childless. In some cases, it is necessary to give birth to male children, as in some geographical areas only male children were considered to be children (*τέκνα*), thus a childless woman (*άτεκνη*) was the one who could not have male children. Petropoulos [77] says that when a woman cannot have children, or has only female children, the husband could replace her and have another woman, and elsewhere in the same paper, he also writes that divorce was justified if the woman was childless or had only female children. Thus, in many areas, until very recently, a woman was considered childless if she did not have sons who were the ones who could be the heirs to the family property. A woman, in this case, was not only childless but also “without heirs” (*άκληρη*). This is associated with the Vedic/Semitic scriptures according to which, the God of gods created the gods (only males) and he passed to them his divine qualities. Thus, it is only the father (God) who can pass down his qualities to the son (heir). Otherwise, his qualities cannot pass to the next generation. This is also behind the surname going from father to son but it is not handed down from father/mother to daughter.

As already mentioned above, the study of the root “τοκ-” as in “*τόκος*= interest, profit gained or *value that a person pays to have what this person wishes to make use of*”. The questions asked here are firstly, what is the gained profit when a (male) child is born? Secondly, what is the value paid and by whom? Finally, what does the woman wish to make use of the situation of having children or male children and in what ways does she make use of them? From the study of the word child (*τέκνον*) but also *γόνος* from the root γεν- used of men, one can see that on the one hand the profit is that the human species in general and the specific blood/family line will be secured, so the nation (genre) will survive. In addition, not only this but as the descendant (*γόνος*), related to brave (*γενναίος*), a male child will be able to fight for and protect the nation, as a brave warrior (compared with the ancient German root *degan* and the ancient Scandinavian root *pegn* or ancient English *peg(e)n*, all meaning “*warrior, servant, young man*”). We could say that one wishes to make use of the (male) child to protect life or personal/family life. Thus, the woman adds value if she has (male) children and her social and family position is guaranteed with all benefits and rights. Otherwise, she is “useless/inconclusive” (*ατέκμαρτη*), or futile, useless, not bringing any results, unprofitable line (*άκαρπη*/*άγονη*), or leading nowhere, ineffective, not creative, not giving any products or results (*στείρα*). Thus, she is defective and there is no place in life for her. Interesting is to see the use of the word infertile (*στείρα*) as the word also implies that it is germ-free. The question raised here is what could be the force that could “pollute” in the case of childbearing? Further study is needed here as to this point and what happens when two get in such close contact, the exchange of information that takes place in this case, the virginity issue, and the need to maintain absolute purity in theology or the need to maintain the purity of the archetype ideas as in Plato and the risks of misunderstandings.

In contrast, a man is the one who provides the information for the new organism (the child) who will continue the human qualities, the nation, the family, the genre (γένος) by being the brave one who will fight for the nation. The sperm is the information carrier. It is significant for the man to have male children as this proves his own ability to fulfill his role or task of protecting the continuation of the nation. The birth of daughters, especially if only daughters come to the family, was considered to be a big humiliation for the man who is unable to participate and fulfil his social role. In case he was childless, this meant that his sperm was not that information carrier and he was also called “άσπερμος: from the suffix α- that denotes absence and sperm” a word used initially in botany or agriculture to speak of plants bearing no seeds, then used only for sterile men who could not produce sperm and consequently could not leave any descendants behind, thus defective and a shameful disgrace.

Furthermore, there is more in this category about the archetypal qualities of the two genders. The man is associated with knowledge as men can “*mentally conceive*”, “*mentally create*”, “*materialize*/*implement or bring something new to the world*” all associated with mental activity. Then, a man is the one who has a brave body and attitude that goes ahead despite challenges and guarantees life sustainability. In Jungian psychology [78], it is the spirit, the light, the truth, and the justice elements, the stimulus that interacts with the chaos, the bearer of all potentiality, the female aspect, and initiates life. Thus, a childless man is one who cannot respond to this. He cannot fulfill his destiny. His mental qualities not being there, he is unable to conceive, create, understand, and know or unable to bring anything to the world. He is defective, incapable, impotent, futile, useless, inconclusive, and a social ridicule.

The woman is associated with receiving the information carried by the male sperm and safeguarding that this information will manifest as a (male) child, as this information is without defects (as in the case of a female child born). In Jungian psychology, the woman is the container of continuity, the maintenance force, the symbol of the earth that can keep, gestate, and support lifeforms. A childless woman is far from the archetype full of defects. In both cases, the word “*ατέκμαρτος*” in the meaning of “absence of perfection” and “having defects”, both somatic and spiritual, is relevant. This aspect is related to religious aspects and will be further explored below. Considering the meanings of infertility in other countries included in the current study, it is seen that similar attributions are made to childless women and men.

Similar in some respects to Greece, in Turkey, the meaning of childlessness for men is inadequacy in masculine functions, inability to satisfy the paternal instinct, being alone in old age, lack of support in business life, inability to continue the lineage, lack of social role (such as not being a father, father-in-law, grandfather) and lack of social security [75]. In addition, womanhood and motherhood are intertwined, so childlessness is a situation that makes women feel more incomplete [79]. Furthermore, in some families in Turkey, more importance is attached to having a son than to having a daughter. According to the patriarchal view, the son was seen as the continuation of the lineage and the heir of the family. For this reason, giving birth to a boy was also important for the status of women and men [80]. In relation to determining the sex of the baby in advance, according to Ziya [81], a woman with more femininity is more fertile. As a woman’s femininity increases, the probability of giving birth to a girl will increase, and as her masculinity increases, the probability of having a son will increase. Women with weak nerves or who are not healthy enough will also have girl babies, and if they want a son, they must first get treatment. A woman who wants to have a son should take plenty of protein, wash with cold water in the morning, increase her physical activity and limit sleep. Ziya [81] supports his suggestions with his study, reporting that seventy-eight of a hundred women who properly regulated their lifestyles and diets achieved childbearing of the desired sex, and only twenty-two failed ([81]; as cited in [13]). Although these suggestions are outdated, today there are some families wishing to have a son and rituals are performed for this aim.

In Lithuania, a family having only daughters was not considered childless, but the birth of a son in the family was considered an honor. According to older people, a boy is a pillar of the house, and a girl is just an expense because she will need to be given a dowry and she will leave her native house. The son is the most important because he is a successor of the family, the heir of the father’s farm [19]. So, if a woman gave birth first to a girl, she would often suffer the husband’s reproach. Besides, a son could become a priest, which was the biggest honor in a family.

It was believed that the couple could contribute to the sex of the baby by performing all sorts of rituals. Gender predictions were also made based on some superstitions [19]. According to Kriauza [18], in the past, it was believed that parents’ temperaments and passion influence gender formation during sexual act. The baby will be of the same gender as the one (woman or man) who would be more passionate during the sexual act. When predicting the sex, people also gave meaning to the age difference of a couple. It was believed that the baby would be of the gender of a younger person in a couple [19]. Based on the principles of magic, it was believed that to have a son, a man had to lie down with shoes and a hat or put a bridle under his head during the sexual act. These beliefs were widespread throughout Lithuania and were recorded in the 19th century and at the beginning of the 20th century [19].

The culture of preferring a boy over a girl was also prevalent in Malta up until the early 1970s. As in other societies, the tradition of inheritance from father to son together with a reliance on boys to provide economic support and to ensure security in old age, were part of a set of social norms that placed greater value on sons than daughters. Through equal opportunities for education and employment conditions, this culture is now almost non-existent. However, the strong Catholic upbringing seems to play a big role in the decision-making process of childless women. Maltese society traditionally views household and childbearing as the sole responsibility of the women, and that has not changed much throughout decades. Childless women, whether voluntary or involuntary, report that pressures are most present in the childbearing years and a sense of relief is present after they pass.

Based on the previous empirical studies on childlessness in Finland, motherhood is often strongly connected to womanhood (e.g., [82,83]). Therefore, childless women may feel inferior [59], incomplete, or incapable of fulfilling the role of a woman [84]. One may even claim that while society may see childless women as losers, childless men are seen as interesting [85]. The feelings of involuntarily childless women are also often related to the way that they see the female body [86,87]. The same way as a woman’s body may be a cause of disappointment and target of treatments for an involuntarily childless woman (e.g., [82]), sterilization may be a way for a voluntarily childless woman to fix her body as “it is supposed to be” [23,88].

Most studies about childlessness worldwide usually analyze women. From a feminist perspective, the decision to remain childless has been described as an expression of a self-determined life, as in previous generations a woman’s life had been constructed around the roles of wife and mother [89,90]. On the contrary, “male childlessness” has only recently started to be studied [91]. In our search, only in Finland we found relatively many studies that focused not only on women, but also on men (e.g., [92,93,94,95]). In Turkey, it was seen that studies on male infertility are mostly carried out from a medical perspective, whereas studies on female infertility are performed from a psychological perspective. A challenge for researchers analyzing childlessness in males in part is because men tend not to identify children from divorced marriages/partnerships; measurement is also complicated by the fact that their reproductive period is longer [35] and less clearly defined [96]. When it comes to childlessness, despite gender, researchers also face another challenge. It is difficult to accurately calculate the proportion of voluntary childless people in the population, as censuses often do not ask childless individuals whether they are childless voluntarily or due to situational reasons [97].

In conclusion, today, we are invited to revisit gender issues and their associated individual or collective/social needs or fears and take into account the contemporary principle of gender equality. Combined efforts need to be channeled to introduce changes in attitude which will gradually heal gender conflicts and polarization as a survival mechanism to protect women from stigmatization, pain, and social/personal distress. This will lead to more gender balance.

#### 3.2.3. Need for Continuation and Survival after Death

In the 2nd century AD, Athenagoras said that when you have children, you continue to exist and you beat death. In his words: “*Ποιείται δε και παίδας επί τω είναι τε διαμένειν καθ’ όσον οιόν τε τους υπ’ αυτού γεννωμένους τη των παίδων και των εκγόνων διαδοχή την εαυτού τελευτήν παραμυθούμενος και ταύτη το θνητόν απαθανατίζειν οιόμενος*” (translation: “And he gives birth to children so that he can live on, as long as all those descendants born, children and grandchildren, are the ones to speak about him, and this mortal is immortalized”) The author of *The Book of Proverbs* says that “The one who gave birth to a son has never died”. In Greek “*Ο γαρ γεννήσας υιόν ουκ απέθανεν*”. In Migne [98], childlessness was the worst evil as “it completely erases the memory of the dead” (Saint) Isidoros Pilousiotis ([99] PG, 78, 1160). In the saint’s words: “*Η ατεκνία μέγιστον κακόν ως παντελώς σβεννυμένης της μνήμης των τεθνεώτων*”. In the Byzantine times, children were considered to be the images of the parents, they continued the family line and resurrected the dead ancestors according to Basilii Seleuciensis. In his words “*Η γαρ των εξ ημών φυομένων παίδων διαδοχή τούτο έστι, τούτο βούλεται, της των σπειράντων και φυσάντων εικόνος εν τοις παισίν αύθις ανανεουμένης αεί και τρόπον τινά πάλιν ανισταμένης ως δοκείν εκείνους τους πάλαι κατοιχομένους εν τοις ζωσί τε πάλιν και περιούσιν ανθρώποις οράν*” (translation: “This is the succession of children born by us and this is what it wants to achieve, the resurrection of those who provided the seeds and made them in the image of theirs so that people can see them again”).

As we have seen in the Greek language, it is the male who provides the information for the new organism (the child) who will continue the nation, the family, the genre (γένος) by being the brave one who will fight for the nation. The nation was secure against invasions or external enemies as the male children were the brave warriors to fight for life. It is still present today, as we still send our young male sons to the army, asking them to protect the national borders or fight for life. Finally, the family with male children was also guaranteed to thrive as the family had more hands to do hard work in the fields, or tend to cattle or industry. Followed by the cultural aspects of the family name being handed down from generation to generation only from the male line, the birth of strong male children also meant the continuation of the specific family genre in the future, a guarantee for eternity. The mother of male children participates in these efforts to provide continuity and survival, and thus her social and family position is guaranteed with all benefits and rights.

In the past, a family with children was guaranteed to survive, as the children were the carers of their parents when they became elderly or weak, no longer able to sustain themselves [99]. This has changed today. Social support systems in the form of pensions, health care, and social services provided by the welfare state [100] have met the old age needs to some extent, despite the sad stories that still echo experiences of loneliness, isolation, abandonment, and death among the elderly populations.

Research on well-being in old age has shown that parental well-being is positively influenced by the presence of adult children [101] and even on mortality; people tend to live longer if they have a surviving adult child, associated mainly with the emotional and social support that is available to them in case of need. This remains the same even when parents have outlived their children, have abandoned, or lost contact with them [102].

As in Greece, having a child in Turkey ensures the continuity of the generation [44]. In the Southeastern Anatolia region, since men are considered as the carriers of the spark of life and the continuation of the lineage, those who are not married or have no sons are considered as blind hearths because there is no one to maintain their hearths [80]. Similarly, in Turkey, parents expect to receive attention, care, and support from their adult children when they get older. This is considered the same among the Maltese society. In the case of women especially, being childless is associated with the fear of death, as the spirit will not be passed on to the next generation, and there will be no one left to recall that childless person after they die. This is even more prevalent among women who have experienced a miscarriage or stillbirth or a failed IVF [103].

Considering this human need for safety and survival, especially at times of adversity, each one of us, together with policy makers, can take responsibility and make those decisions or adopt behaviors which will free future generations from the role of acting as the ones who will safeguard life for parents and grandparents. This is because these goods will be safe for all, so that parenthood can be inspired by the higher ideal of co-support for the co-evolution process, heralding a new environment signal sent to the children to be born.

#### 3.2.4. Religious Attitudes and Beliefs

Some of the attitudes also come from religion. Until the middle of the 19th century, infertility was considered a punishment from God [104]. The Christian approach still considers the birth and upbringing of children to be the most important task of marriage [105]. In Christian scriptures, infertility in the family is considered the greatest heavenly punishment and shame [5]. Since most Lithuanian residents are Christians, those beliefs affect the perception of childlessness in a family.

In Greece, according to the orthodoxy, the purpose of marriage is to have children (*τοις δε γεγαμηκόσι σκοπός η παιδοποιία*) as Klimis Alexandreas (Παιδαγ. 2, 10) said [106]. As childlessness was the worst evil, it could also be considered to be the result of sins [107]. Although the predominant religion in Malta is Catholicism, the same stands true in Malta.

Turkey is a secular country where many religions are practiced freely. However, the mainstream religion is Islam. It should be kept in mind that both Islamic practices and the level of religiosity—regardless of which religion—may differ from person to person. According to Islam, the institution of marriage and having children are considered important. Childbirth is perceived as a divine reward, while infertility is perceived as divine punishment [108]. The religion of Islam sees infertility as a trial of God and treats it as a disease that needs to be treated. However, techniques that involve the combination of any sperm or egg extramarital are illegitimate. For this reason, some assisted reproductive techniques cannot be preferred by Muslims. According to Islam, it is appropriate for couples at the beginning of their marriage to delay having children for a certain period of time by agreement of both spouses and to use birth control methods that do not include a permanent procedure on the body. However, we could not find any Islamic information related to the choice of lifespan childlessness. Another issue that is highly related to religion is abortion. There are different opinions among Islamic jurists regarding abortion and in what situations and until what week the fetus can be taken. However, the verses prohibiting killing and the available medical data make it necessary to say that it is not permissible to interfere with pregnancy from the moment the sperm combines with egg [109]. From this point of view, it can be assumed that the level of childlessness among Muslim women is lower than that of secular women in Turkey.

Religion (mainly Lutheranism) does not have a very visible role in today’s Finland and Finns may be described as rather liberal and secular [25]. Religion may still influence people’s lives, for instance by having strong opinions about the ethical aspects of fertility treatments [54]. In addition, Finland’s increasing multiculturalism leads to a richer variation of different religions, meaning that religions may have a different impact on childlessness in the future [86].

This paper brings attention to the role of influential bodies that shape attitudes and behaviors of health and/or disease. Religion being one of them, especially in the past. Yet, religion is not the only attitudinal influencer. It is the duty of those who have the power to influence populations to make sure that the information is cleansed so that it adds quality to life as well as years to life.

#### 3.2.5. Social Aspects (Positioning, Roles, Social Structures)

In Greece, childless women were considered to be sinners, bad luck bringers, and useless (*Άκληρη* (=without heirs), *στείρα* (=sterile/infertile), *μαγκούφα* (=thick-headed person to be avoided, without family, all alone, miserable), *μαρμάρα* (infertile, sterile, bad), *γρουσούζα* (bad-luck bringer), *ανεπρόκοπη* (=useless), *βάσκανη* (=with an evil eye), and *αμαρτωλή* (sinner)), thus they were stigmatized and avoided, never invited to participate in social ceremonies, weddings, or christening rituals. When they went to church, people looked in the other direction not to see them as they had the evil eye. The priests did not accept their offers to the church. They resorted to whatever they could think of to save face and avoid being marginalized: witchcraft and acts of prejudice, fasting, prayers, herbs, oaths. It also showed in the names of girls who were born but not preferred, such as Agoro (=boy), Stasini (=stopping), Stamata (stop), so that the birth of girls could be controlled and only boys to be born. Sometimes, women went to extremes by pretending to be pregnant when their husbands were sailors and during their absence so that they could adopt a child to save their social dignity. Childless women were also disgraced within their own families as well, besides the wider social context [110]. We have already mentioned the phenomenon of synkria or synkormissa, second woman not necessarily wife, with the aim of getting a child in areas such as Mani but elsewhere in Greece as well.

The wish was for the birth of a boy to continue the family name. Especially in a royal family, the birth of a boy meant succession to the throne and it was celebrated. This is evident even in modern history, in the case of Napoleon Bonaparte, who when he became the emperor and his wife Josephine could not bear him children, he remarried a younger wife, Marie Louise of Austria to bear him a son.

Females were considered to be the weak gender and that is why in Mani (also elsewhere) the birth of a male child was greeted with gunfire celebrations and other joyous festivities, good wishes for him to become a brave warrior to push enemies way back, while the birth of a female child was considered to be the second bad luck in the life of a woman (the first being that of being childless). When asked what kind of child she gave birth to, the answer was “the good kind” if the child was a boy and “girl” when the child was female, implying that girls are not the optimum quality. Phrases such as “the pregnancy was worth nothing” in the case of a girl being born emphasize the status of both women as a gender but also the perceived failure of the woman to meet the social expectations as the bearer of sons, classifying her to an inferior social status. Female children were a disaster and the curse of the family. No matter what the financial status of the family was, parents of girls had the same agony to get their girls married with whoever, offering a dowry to the groom. That would be easier for a rich family but not for a widow or a poor mother who had to work hard to get enough for the dowry. However, they also wished for the first child to be a girl to look after all other boys to follow and parents when they get elderly.

Finally, the woman who could give birth to boys was alienated from her own maiden family and entered the household of the husband that was controlled by his mother. Thus, the woman had to be like her mother-in-law, otherwise, there would be no survival for her. As for the opposite, that is a childless woman, she had no place anywhere, ran the risk of death, or was abandoned to survive alone, which was a terrifying option. The adopted children were treated not the same way as the biological children (cases of orphans with no relatives). Adoptions of children not related in any way were rare until the beginning of the 20th century [10].

This paper offers an opportunity for all of us to reflect and become aware of the means societies use to obtain goals. The old way of punishment and reward either on a personal level or a collective level causes a lot of pain associated with isolation, stigmatization, labelling, and burdening those in dissonance with the accepted norms. It is time to reconsider and provide new insightful, life-promoting solutions to old challenges. History has taught us that the suffering of a group can never be a factor behind civilization and the Aristoteleian happiness for all.

#### 3.2.6. Rights

Due to the poor social position of childless people, they resorted to solutions such as other forms of childbearing, adoption, foster parenting, surrogacy, and IVF being among them, depending on the historical time and the availability of the solution. However, this has not always been easy, even today.

The reality in Greece is that society is still quite traditional and there are aspects that remain to be regulated by legislation. The existing legislation does not fit with the current picture of Greek society. On the one hand, there is a tendency to protect the traditional Greek family bond, and on the other hand, it attempts to impose foreign processes within Greek reality [111].

Greece consents to artificial reproduction for cohabiting couples, married couples, or unmarried or single women. IVF is financially supported by the National Health system and there are many clinics both state and private ones where people can go. Law 4538/2018 simplifies adoption procedures and the registering of orphaned children by eliminating many bureaucratic steps facilitating prospective adoptive parents and promoting foster parenting, while at the same time adding important tools for child protection. Work remains to be done concerning same sex people who would like to adopt or become foster parents as this is a new phenomenon.

In Finland, the current family policies are often too stereotypical and old-fashioned [94], and even though society may see that everybody has a right to parenthood and infertility treatments, everybody may not be equally able to use these services, such as gay couples [112]. As described by Riskilä [107], the government does not cover infertility treatments for lesbian couples and gay male couples may be even in a more unfavorable position. Surrogacy and infertility treatments are not allowed in Finland [112]. Finland created its first adoption law as early as in 1925 [113] and gay couples are allowed to adopt [114]. However, as noted by Moring [114], prejudices may make it difficult for gay couples to actually be chosen as adoptive parents.

In Lithuania, the conditions of assisted reproduction are established by the Law of the Republic of Lithuania on assisted reproduction [30]. According to it, assisted fertilization services and/or gamete banking services are legal, but surrogacy is not allowed. The organization and procedure of adoption is defined by the “Description of the Organization Procedure in the Republic of Lithuania” [31]. Same-sex marriage or civil union is not legalized in Lithuania, so there are no legal possibilities to have a child in that union.

In Turkey, assisted reproductive techniques can be applied in institutions authorized by the Ministry of Health [115]. According to the philosophy of pronatalist policies, which identify the female body with fertility, the female body does not belong to women, but belongs to the state, and therefore it is an area where political will and medical knowledge can intervene and shape [13]. Women become subjects as long as they become pregnant, give birth, and raise children who conform to defined ideals. Infertile women are seen either as obedient patients who need to be treated by doctors or as victims who pay for their mistakes [13]. When it comes to infertility treatment, the implication that the rich have the right to want children and the poor can receive the infertility treatment as long as it is given to them creates a knot in the field of family planning [116]. On the other hand, it should not be forgotten that individuals have the right not to have children. In a qualitative study conducted in Turkey, it was found that women who choose to remain childless oppose society’s construction of femininity through motherhood and see motherhood as a lifelong sacrifice that takes away something from their own lives. The participants stated that they see parenting as a role that is imposed only on the mother rather than a shared task between women and men in society, and they prefer to remain childless because they do not want to take on this role [117].

In Malta, the Roman Catholic religion is the official religion, as cited in the second article of the constitution [118]. Assisted reproductive policies are regulated by the Embryo Protection Authority, and are regulated by the Embryo Protection Act [119,120], as amended in 2018 [121]. In the previous two decades, IVF had been practiced in the private sector in Malta in spite of there being no law. The sorry plight of infertile couples has attracted the interest of local obstetricians and gynecologists. In private practice between 1969 and 1975, the records of one gynecologist show that 332 couples sought advice about their infertility, an average of 47 per year. According to Camilleri [122], “Yet so many of them seemed to become readily disheartened, in some cases because they expected “miracle” pills or injections, in others because the husband would not countenance the idea that he should be investigated, and in many cases for no clear reason at all”. Once the law was enacted in 2013, services for assisted reproductive technologies also started in the main public hospital. These services were initially partially free, as the patients had to buy the medication themselves, but from 2022, even these medications will become free for Maltese residents [123]. Heterosexual couples in a stable relationship, as well as same-sex couples and single women, can access IVF services in Malta. However, surrogacy is still not allowed.

In conclusion, we should bear in mind that decision making should consider not only the wisdom gained from the past but should also project the consequences of whatever current decision making may have for the many generations to come. A lot remains to be studied concerning the ethical but also the biological or psychomental and social issues associated with technological interventions as adaptation mechanisms for childlessness for the babies born and the quality of societies to be seen.

#### 3.2.7. Cultural Aspects

Culture plays a significant role in how childlessness is perceived in a society. Motherhood is still a norm in many cultures and childless people experience social pressure [37,54,59,82,85,86,87,88,124,125,126,127]. In Greece, being childless is like a “dengue fever” experience [128]. The movement of childless and childfree, which seems to gain ground over the last years abroad, in Greece still stumbles on raised eyebrows and smiles of sympathy. Accordingly, partly negative stereotypes and negative perceptions towards voluntary childless women exist in society [129] and this applies to all participating countries. For instance, they are seen as being selfish, unable to settle down, irresponsible, hating children, unnatural, and incomplete [23]. They may also be perceived as “missing”, “half”, “undesirable women” and are marginalized [129]. Furthermore, people may see them as not fulfilling the duty of womanhood, being unaware of the sanctity of motherhood, having difficulty in communicating with children, not loving children and, regretting at later ages [129]. Finally, a set of contrasting adjectives, such as altruistic/selfish, acceptable/marginal, sacred/worthless, also get attributed to such women [27].

Yet, the childlessness phenomenon is becoming more common in many countries, such as Turkey, especially among the young, educated, and middle class, although there are no networks organized by these women [36]. However, while society approaches infertile women with sympathy-based feelings and does not judge their childlessness, the medical and normative literature on infertility shows how uncertain and transitive the boundaries of the personal and the political and the private and the public are in the context of late Ottoman society [13], and not only there but in other countries as well, which introduce pronatalist policies encouraging people to reproduce [93,95]. In a study conducted in Turkey, it was found that participants with more sexist attitudes had more negative prejudices against voluntarily childless women [130], while, in another study also conducted in Turkey, individuals who are parents were perceived as warmer and happier in their marriage than those without children, who were evaluated as more determined but more emotionally problematic [131].

Childless women may threaten the role division that the society so strongly tries to maintain, and therefore they are pressured to either have children or at least to have an opinion about having children [23]. That being said, society’s attitude is changing and different life courses are becoming more accepted in modern western societies [87,94]. During the last decade or two, voluntary childlessness has become a more common and regularly discussed phenomenon in many countries, such as Finland [93]. Voluntary childlessness can thus be seen as a consequence of cultural change.

According to Folbre [132], raising children requires the investment of substantial financial and time resources by parents, and there is a general recognition that the costs associated with parenthood outweigh the benefits, at least while children are young. As cultural values seem to change and money is considered in decision making, it is no surprise that there is a percentage of people who measure the value of having or not having children by taking into account the cost of raising them. However, even looking at this, we will still see that the same core issue exists: fear for survival, only that now the survival lies with the one who has money.

Choosing childlessness brings to the forefront new attitudinal issues and initiates new discussions over facilitators and barriers [133]. However, despite the arguments brought forward in support of a decision for voluntary childlessness life experiences, a more analytical examination of the driving forces behind such decisions will reveal deeply seated primal traumas of rejection or neglect or abuse which remains to be healed and which may be behind communications that speak about the absence of good reasons why we should bring children to the this futile, cruel world, the benefits of pursuing personal goals, dyadic happiness, academic or career recognition, equality, not sacrificing personal life experience for children/others, etc. A careful analysis of the narratives can reveal unmet emotional needs, identity issues, being seen as social/familial capital or even a commodity, relational challenges, injustice, pain, fear, and anger. Perhaps, the movement of antinatalism, both in modern times initiated by the German philosophers Arthur Schopenhauer and Philipp Mainländer and in antiquity [134], is a pain in disguise. As Sophocles says in the chorus: (1225) “Not to be born is, beyond all estimation, best; but when a man has seen the light of day, this is next best by far, that with the utmost speed he should go back from where he came. For when he has seen youth go by, with its easy merry-making, (1230) what hard affliction is foreign to him, what suffering does he not know? Envy, factions, strife, battles, (1235) and murders. Last of all falls to his old age, blamed, weak, unsociable, friendless, wherein dwells every misery among miseries”. Research remains to be made as to the deepest issues at work behind antinatalism.

Therefore, although attitudes towards childlessness are changing and discussions about voluntary childlessness appear more often in societies, changes do not come as quickly in some countries. For example, the model of childless families is a universally unacceptable phenomenon in Lithuanian society, and voluntary childlessness is rarely discussed because it does not correspond to the widespread concept of the nuclear family [37]. Having children in society is recognized as the norm and almost a necessity and large families are often considered to be the ones who “protect” the Lithuanian nation from extinction [37]. Nonetheless, due to more frequent people’s rights movements during the last years, the situation is getting more positive.

It is high time to revise priorities, roles, values, principles, and philosophies so that we can move from culture to civilization. This will enable the transformation of the environmental signals from that of fear and pain to the new one of love and compassion, creating health, good relationships, peace, and civilization, away from medieval dark experiences.

#### 3.2.8. Folklore

According to ancient Greek mythology, Gello was a young woman who died a virgin and returned as a ghost to do harm to the children of others [135]. The word comes from the root γελ- from which the word laugh (γελώ) is derived. It is associated with the laughing, ridiculing face of Gorgo who spread death to whoever looked at her and who is considered to be the origin of reproductive demons. The ancient Greek poet Sappho wrote the phrase “(she is) fonder of children than Gello” (“Γελλώ παιδοφιλοτέρα”) [136]). Gello is also associated with 1. *Gallû* the Babylonian, an Assyrian demon who was believed to bring disease and death, or 2. The *Abyzou* [137] (etymology abyss), another female demon, who was motivated by envy for she was childless herself, attacked women, and was blamed for miscarriages and infant mortality. In the Byzantine years, the plural form of Gello “gelloudes” is met to speak about the demons similar to Stryggai, the birds of ill omen who fed on human blood and flesh and who related to witches and malevolent folkloric beings [106], as cited in [138]). So, gelloudes, like other demonic beings, entered houses at night and strangled infants or caused the death of pregnant women and their babies in gestation. Gello/gelloudes are also associated with crones and hugs, the envious, old, malevolent female folklore figures of sinister nature and supernatural powers who could cause problems to women and children [139]. Such associations can explain the fear of society projected onto childless women, who were visited by gelloudes, demonic figures, crones, hugs, and other malevolent beings, and thus they were “polluted, contaminated” with the evil eye and had to be avoided or marginalized to escape consequences.

In the Turkish epics, childlessness is fictionalized by tradition before the child is actually born to emphasize the importance of the epic hero. The “childlessness motif” is considered as the defining feature of the hero to be born in the whole Turkish epic tradition. The mother and father’s episode of childlessness ends with the birth of the “hero” baby [45]. In another study focusing on the rituals of healing childlessness, in Turkish folk beliefs, it is thought that the spirit of death haunts families who constantly lose their children [140]. Many healing rituals have been practiced since the ancient Turks with the aim to have a child born healthy and live a healthy life. As a result of this way of thinking, a ritual based on the principle of indirectly possessing the child and hiding the truth from the spirit of death is practiced in Turkish communities in general. It takes the form of metaphorical selling–purchasing in order to keep others alive after the deceased child. In such rituals, the childless woman says “I sold my baby to you” to a woman with a healthy child, re-enacting the moment of sale among women, dressing a healthy baby for a baby at risk, leaving the baby somewhere and then taking them back can be examples of these metaphorical sales rituals.

In a study aimed to investigate the relationship between childlessness and the figure of the snake in Turkish tales, childlessness is dealt with predominantly by women [44]. Women beg God to have children and state that they risk everything to change their fate and to be accepted both in their marriage and in society. They are willing to have a child, even if it is a snake. In some tales, the hero of the tale is the sultan, who seeks remedies for childlessness and begs God to end the situation they are in. As a result of his prayer, he happily accepts the snake (child) he has and embraces it. In these tales, it is emphasized that childlessness is not subject to status and that childlessness is negative for everyone. In some tales, childlessness is seen as a problem for both women and men, and the expression of family or couple who want to have a child is included in the narrative. In these tales, it is emphasized that childlessness should not be handled individually by a woman or a man, but that being childless concerns both spouses. In tales, the prayer of the husband and wife who seek remedies because they are childless and pray to God to give them a child, even if it is a snake, is accepted. In tales, with the figure of a snake, it is told that having a child is a great blessing and one can risk everything to have a child. In the tales that mirror the value judgments of the society, the importance of having a child is emphasized by considering that childlessness is an indicator of deficiency and fault for individuals [44].

In Lithuanian folklore, since pregnancy was expected in any marriage, hundreds of superstitions related to the fertility of newlyweds are found, and many rituals were made before and during a wedding ceremony. According to Paplauskas-Ramunas [17], the purpose of these numerous superstitions was for the bride to invoke a blessing of a large family. One of the superstitions performed before a wedding was for a suitor to swallow a raw egg so that the future husband would marry a fruitful woman [18]. The mother of a bride would gather all the crumbs from the table and feed them to a pregnant cow for her daughter to become pregnant immediately after the marriage [17]. The bride also tended to put on her blouse or dress the wrong side out for the same purpose [141]. Both the bride and the groom circled the table three times in her home, and the number of times the bride’s maids touched the tablecloth indicated the number of children the couple would be blessed with [17]. To get pregnant, women also drank special kinds of teas and prepared baths from many kinds of herbs [17].

The pagan Lithuanian women asked the Goddess of Luck—Laima, before the birth. A special drink was also prepared in gratitude for the Fertility Goddess—Žemyna in gratitude for having blessed the birth. The snake was also thanked for the birth of a child, and a couple made sacrifices for the snake in pagan times to conceive a family [142,143]. Snakes in Lithuanian mythology, tales, and legends are associated with the beginning of life. Even on a linguistic level, the word “snake” (gyvatė) is related to such lexemes as “life” (gyvybė, gyvenimas) and “alive” (gyvas). It was believed that if you dream of catching a snake in a dream, it means pregnancy and the birth of a son for a woman [143]. In folklore, the snake is considered the guardian of new life, and man must not drive it out of the house or harm it.

In Finland, the role of a mother can still be seen to be very much influenced by the oldest cultural stories on motherhood and myths about the Goddess and Mother Earth [60]. Early beliefs and superstitions on fertility and childlessness differed substantially between the different regions of Finland, and they were sometimes also contradictory [21]. Yet, the cause for infertility was often seen in a woman, who had committed a sin or “had become eyed”. To ensure fertility, one ought to marry young, live without committing sins, and avoid being “eyed by a bad eye”. Moreover, various spells were believed to improve fertility [21].

Paulaharju [144] presents old habits and beliefs about birth, childhood, and death in eastern parts of Finland. According to him, married women who were not able to become pregnant were not appreciated, and they were compared with infertile animals. He noted, however, that God has, on the other hand, given a good part for these women, who do not have many children, because they are able to be like animals every once in a while. With “to be like animals” we assume him to refer to the carefree life of animals. Paulaharju [144] does also share some tips on how couples can successfully get pregnant: married couples need to heat up a sauna and have intercourse there. In addition, women suffering from infertility can turn with their prayers to their relatives, who have already passed away. Yet, if a woman has married against her parents will, any prayers or spells will not work. Accordingly, childlessness may be caused by marrying against one’s parents’ will [144].

To conclude, according to folklore themes in different countries, it could be stated that childlessness has been a challenge for humankind from the very beginning. That is why the folklore motifs tend to be deeply rooted in the universal consciousness and are found in so many different countries. Folklore, myths, tales, and stories of this kind seem to have been used to prepare younger generations to adapt to social circumstances throughout history. The question is, are we ready to take the next step?

#### 3.2.9. Media

Researchers find that the image of parenthood is still fully supported, and individuals, especially women, who have not had children are underestimated and often stigmatized in the media [145,146]. In foreign media, the debate about women’s voluntary infertility began at the end of the 20th century [147]. After analyzing the British media, Giles et al. [147] found that between 1990 and 1995 the topic of voluntary infertility covered about six percent of all papers about infertility. The articles about voluntary infertility received more negative reviews than those about involuntary infertility related to physiological reasons. LaMastro [148] found that voluntary childless individuals in the Western media have generally been described as immature, selfish, and cold.

The topic of voluntary infertility in Lithuania appeared around 1991–1996. The topic was not a direct one but you could read the message behind the lines. Sex education of adults and adolescents was associated with postponement of childbearing or temporary infertility [149]. In addition, it was perceived as a tool that could do more harm than provide benefit. Many of the articles recognized a woman’s purpose as a mother as a savior of the whole nation. Abortion issues were associated with the risk of ultimate infertility and were identified as a major risk to a woman’s reproductive, mental, and physical health in Lithuanian media in 1991–1996. The use of modern contraception was also associated with an abortion. Besides, abortion was associated with other social problems—a sharp decrease in the country’s birth rate, an increase in the divorce rate, and the sexual freedom of young people are also associated with the prevalence of prostitution and venereal diseases [149]. Childbirth was also mentioned as a method of preserving beauty and youth [149].

In general, expecting a baby in Europe—and probably all civilizations until recently—has been an integral part of the perception of love and beauty [105]. Although researchers disagree on which female facial feature is perceived as the most charming, many studies have found that when a group of men are shown photos of women’s faces, they choose the faces of those girls who are currently in the middle of the fertility cycle as the most beautiful [150].

A woman’s infertility was stigmatized much more strongly than a man’s in the Lithuanian media in 1991–1996 and her single life was presented as a state that every effort must be made to avoid. The refusal of having children—not because of material deprivation—was explained by the “immorality” of single mothers; they were called “cuckoos” [149]. Nevertheless, during the past few years, some positive articles about voluntary childlessness, a person’s right to decide to have children or not appeared in the popular media in Lithuania. It was also related to such causes as helping to reduce climate change, supporting human rights, etc.

In Turkish media, there is a lot of content on many different topics related to infertility, pregnancy, pregnant women, and mothers. On the other hand, voluntary childlessness in Turkey is a phenomenon that does not find many places for itself in the popular media, is not discussed with heartwarming discourses when it does, has a weak voice on some alternative websites and blogs, and is included in academic studies only through the translation of Elizabeth Badinter’s and Corinne Maier’s books [36]. According to a study by Onnela [151], the Finnish media, in turn, nowadays talk more positively about childlessness, emphasizing the importance of individual rights and values.

In Greece, the media are harmonized with the culturally prevalent and already discussed ideologies concerning the nation, gender, motherhood, and personhood, and the newspapers until recently, but also the other media channels over the last few decades, simply mirror reality and legitimize dominant discourses about how persons, especially women, ought to manage their fertility, exalting motherhood and implying disgrace for anything other than that [152].

In Malta, the topic of infertility and assisted reproductive technologies began to be openly discussed in the media around the year 2010, which then led to the establishment of the first IVF law in 2012 [120] which was then further revised by the Embryo Protection Act [121]. Prior to this, although IVF services were available privately, the discussion of the topic was somewhat more of a taboo [153]. Nowadays, there is certainly a more open discussion and awareness around infertility and involuntary childlessness, including following miscarriage and/or stillbirth, with the aim to assist bereaved parents who feel alone and isolated from society. Research has been in a Maltese context as a means to understand how cultural and social structures mediate and even complicate couples’ psychological functioning and wellbeing, especially in the case of the female counterparts [103]. This is important to pave the way to a deeper understanding for counselors to effectively assist clients when they seek support for experiences of childlessness.

This analysis is also a call for health professionals involved to integrate transcultural skills so that they can better understand what is communicated beyond words. This better transcultural competence together with transdisciplinary knowledge can hold the key to offering health services to the people that come to their practice. The call for “successful” conception may not be a call for that per se but a call for healing a deeper trauma. Getting to know media representations and exchanging mono/dialogues can reveal more about the real nature of the narratives heard in health spaces.

#### 3.2.10. Demographics

In all generations, the number of childless women with high education is bigger than those who have given birth in Lithuania. Besides, in the capital and big cities, there are more childless women (as much as 10% more than in smaller towns or villages). As well as in all generations, a much higher proportion of childless women than women who have children have never been married [154].

According to Eke [36], in Turkey, childlessness is often an alternative for middle-class women. However, it should not be inferred that every middle-class woman can easily choose childlessness. It has been stated that women could evaluate whether or not to have children as a choice only through various motivational elements and real conditions. One of the most important components that make this choice possible is feminism. According to the findings, women who choose childlessness most freely make feminism part of their identity [36]. In the same study, it can be said that the preference for childlessness is more common among women living in metropolitan areas, free from family, relatives, and neighbor networks, compared with those living in small-scale settlements with more closed and close relationships and primary family members [36]. Similarly, it has been observed that women who voluntarily choose childlessness in Turkey are educated, have a profession, are active individuals in the social and economic fields, and that these women have an extremely high awareness of the society they live in and of themselves [129]. Moreover, considering women applying to the IVF center, the level of women affected by infertility increases as age, duration of the marriage, and time of wanting a child increase [155]. Women who are primary school graduates, do not work, do not have social security, and have a low income are more affected by infertility [155].

Finnish women and men are among the top three European countries when it comes to the lowest fertility rates [25]. As in many other countries, also in Finland, the average age of giving birth has been increasing, which has led to a decrease in fertility rates [156,157]. In fact, Finland’s childlessness levels are increasing more rapidly than in most other European countries [25]. Various factors may explain the increased age of giving birth, such as education level and residential area, since higher education level and living in a city have been found to relate to a higher age of giving birth [158]. However, higher education levels cannot explain the high childlessness rates, because as noted by Rotkirch and Miettinen [25], in recent years it is in fact lower education that is associated with childlessness in Finland. A lack of a partner may also be one factor behind the increased age of giving birth, and the related increase in childlessness rates [25]. Yet, giving birth at an older age is visible also among people who live in a relationship [158], which suggests that having no partner can neither explain the increasing childlessness rates in Finland.

Finally, the decline in fertility in Greece (as shown in Figure 1 and Figure 2) has been primarily driven by a trend among young adults below age 30 toward postponing childbearing until later in life, as they put a priority on completing their studies and securing a profession [159]. After the onset of the economic crisis, successive record-low numbers of births were registered annually. However, further research needs to be done in the field to better show the link between demographics and childless people.

More research remains to be made to reveal the hidden forces of such demographics connected with childlessness. This paper brings attention to this aspect as well and wishes to inspire researchers to undertake further research.

### 3.3. Development of Different Cases of the Concept

#### 3.3.1. A Μodel Case

The following narrative comes from a childless woman (A) and is a monologue shared on social media: “Well… I feel mad! They treat us as if we were I don’t know what… My sister-in-law and my brother-in-law have been avoiding us for quite a long time though we all live in the same city. We did not know the reason why. But yesterday everything got clear. We learned through friends that she is pregnant. They were afraid that we would bring them bad luck! … They did not want anything bad to happen to them or the baby because we did not have any children. My cousin is also pregnant and we learned the news from a Facebook post! Everybody knew about it and nobody told us a thing! Why? Why? I have never put my nose in anybody’s life, I have always been helpful and kind, I am not a hypocrite. Why are there such bad people? Why do they treat us as the miserable ones”?

The above narrative summarizes well the main attributes of being childless. Even today, we still see the element of social avoidance, the prejudices, fears, pain, and losses. The same is true of the following case. It comes from a childless woman (B). She says: “It goes without saying that there is a lot of social pressure. What is acceptable is for the couples to have children. Deep in me, there is this perception that I will be complete and whole only when I have children. When you are part of the society, you wonder why you are so different. And this hurts… I do not consider myself sick, but the others see me as sick. When a married woman cannot have children, she is labelled sterile and they take pity on her. Society treats people with conception challenges as maimed and miserable”.

#### 3.3.2. A Borderline Case

The case of (C) is a borderline one. (C) is a migrant woman in her early 40s. Her husband died young in a car accident just months ago. Her loss and bereavement were so deep that she could not stay in her hometown where everything reminded her of him and the lost family dream. When she moved to her new country, she fought day and night to establish a new life for herself. She wished she had a baby but she did not manage to get pregnant during the 5 years she spent with her husband. In her country, she had to face the nasty comments from the community and she never felt that she was welcome. Even her sisters, all with children by then, kept being nosey and bitter. She married older than usual and in her mid-thirties and five years into her marriage she was open to having a child, but it did not happen. She received questions such as “when will you have a family?” or “why don’t you have a child?” or “what is wrong and you don’t have a child yet?”, as if they were not already a family and did not wish for a child. But what could they do? In her new neighborhood, she avoided having friends and she kept close to herself in her work environment. It took her months to realize little by little that the new community was different. They accepted her as she was, without making her feel different or ill at ease. Her colleagues invited her to the birthday parties of their children and treated her as one of them. Two months ago, C got a promotion, started psychotherapy sessions to heal the loss of her husband, and now she is more optimistic that life can be better than she previously thought.

Her case is a borderline case, as it has some of the main attributes of the concept but not all of them. There were the elements of stigma and the societal pressure and the pain but in her new environment the attitudes were different, thus alleviating or even negating the burden of her being and feeling childless.

#### 3.3.3. A Related Case

The following story is the case of a childless woman (D). She is in her late 40s, married but with no children. She says: “I have never wanted to have children. On the contrary, my fear was to become pregnant, and then what? I don’t want to live the life of my mother. She raised 7 children and she has been pregnant almost all her life. She never had a life of her own, just an endless duty to take care of us. And we were not happy at all. I was not happy as a child or as an adolescent. My mum was never there for me. She was always busy doing the housework and the laundry and the cooking. I never remember having an interesting conversation or a fun time. She was always tired and exhausted. I do not wish that for me. I want to travel and see the world and I want to study and get involved with interesting things. I want to live my life. As for children? If the worst comes to the worst, if I cannot conceive myself, there are so many other children born and abandoned. I will become a foster mother. I have already had two abortions, my last one 5 years ago. Of course, I am on the pill. I love children and I wish the best for them. It’s just that I do not wish to end up like my mum or them have the miserable childhood I had”.

This case of voluntary childlessness is related to this concept being analyzed but it does not contain the critical attributes. There is a common end, meaning that in both cases there are no children born, but in this case there is an unhealed traumatic experience behind this decision of voluntary childlessness. Certainly, the main attributes are missing here.

#### 3.3.4. A Contrary Case

The case of (E) that follows is a contrary case. (E) is in her 50s, happily married for the last 34 years. She has a Ph.D. and a child. She says: “I have never tried to control life. I have been open to children and I was ready to welcome them. Our house has always been open to the children of the provincial town we live in and all extended family children love spending part of their holidays with us. Anyway, I have always worked with children as a pediatrician and I have developed excellent relationships with both themselves and their parents. Being open to children, I have never been on the pill and I would never abort a child. It just happened. Neither of us wanted to control the birth of a child. We have never seen children as our continuation. We just hold responsibility for our daily needs and experiences. We see children as equal beings who have their own path to walk. And we have crossed our path with that of many many children so far. We have a deep trust in the wisdom of life and we accept whatever it holds for us. It is fine if there are children and it is fine if there are no children”. This final case is an example of a contrary case, as it contains none of the attributes examined above.

### 3.4. Antecedents and Consequences

#### 3.4.1. Antecedents

At the end of the reproductive age, most women may not have children because they are not married or for physiological reasons. Women of childbearing potential do not have children due to delayed childbirth or partner disagreement, fertility disorders, or voluntary decision not to give birth [160]. Moreover, living according to a non-traditional family model, lack of legal regulation of relations in the country (e.g., the inability to marry or formally marry), the financial responsibility of raising a child, and the uncertainty about the future if one has to raise a child alone, also deter a woman from planning to have a child [160].

Childlessness is also compounded by the postponement of the first child, which increases the likelihood that the individual will eventually adjust, become accustomed to the childless lifestyle, until finally losing the desire to have children altogether [105]. Some other reasons are related to contraception, women seeking higher education and gender inequalities in the labor market [153,161]. Cultural norms to have children in marriage have a strong impact on both women of childbearing age and younger women [160].

The risk of developing infertility is increased by the following factors: congenital or acquired genital disorders, previous prostate surgery, infectious and sexually transmitted diseases, toxins, malnutrition, starvation, ionizing radiation, harmful habits (smoking, alcohol, drugs) and under- or overweight [162,163,164,165]. Alcohol and smoking have negative effects especially on sperm functions [165,166].

One of the main causes of male infertility is spermatogenesis disorders that affect sperm quality or quantity [164]. Environmental stress, gene mutations, and chromosomal abnormalities could all affect biochemical events during spermatogenesis, which can lead to abnormal chromatin structure that ultimately leads to infertility [167]. IVF failure and recurrent pregnancy loss are also common in people with DNA-damaged sperm [167].

The main antecedent to childlessness is the inability to conceive and give birth to a living child after a specific time following the start of trying to conceive. According to the WHO [2], infertility is a disease of the male or female reproductive system defined by the failure to achieve a pregnancy after 12 months or more of regular unprotected sexual intercourse. In addition, infertility is defined as primary or secondary. Primary infertility is characterized by no pregnancy that has occurred before; secondary infertility is characterized by at least one pregnancy that has occurred, whether or not it resulted in a live birth [2].

When considering how long it takes to identify a person as childless, it may be necessary to evaluate this time length on two levels: individual and social. The time required to define oneself as childless may begin as soon as the individual realizes that they are not able to have a child, or it may take years depending on the person’s acceptance or denial. In a study conducted among male infertile patients, one of the participants stated that he never thought of himself as an infertile man and did everything the doctors said, while another one stated that he did not perceive the diagnosis of infertility as a disease or an insurmountable situation [168]. In fact, when it comes to voluntary childlessness, this period may not even require attempting to have children. A person may choose to describe oneself as childless from the moment this decision is made. At the social level, it can be effective to have information about both the duration and whether the individual wants to have a child in order to define an individual as childless. For example, some of the couples who were married for a similar period of time may qualify as childless, while others may not. On the other hand, as the duration of marriage or cohabitation without children increases, the individual/couples are more likely to qualify as childless. Another social point is that depending on the importance given by society to the child in the formation of the family, the time for individuals to be identified as childless becomes shorter.

#### 3.4.2. Consequences

##### The Impact on Psychological Well-Being

Childlessness, involuntary in this case, may have a significant long-lasting impact on both women and men, as being childless has become a part of their identity [60]. As described by Lehto [169], “becoming a mother after long-term involuntary childlessness is a rather lonely and merciless process”. Prior childlessness may thus also influence how people experience pregnancy and parenthood [59,60,86,169]. Therefore, it is understandable that infertile women have negative self-perceptions due to their inability to have children and the meanings given to childlessness [125]. The quality of life of infertile women may also be lower [170] because they cannot have children, attending many IVF procedures can tire of it; and the procedures and failures affect their quality of life quality. In addition, women who cannot have children may experience problems such as exclusion/ostracism, infidelity, divorce, or being threatened by their spouse with a second/fellow wife [171]. Accordingly, childlessness potentially causes severe psychological symptoms, such as anxiety, depression, guilt, sorrow, anger, bitterness, loneliness, disappointment, insecurity, fear, exhaustion, pressure, stress, and frustration [60,62,83,84,113,172,173,174,175,176,177,178,179,180,181].

Due to psychogenic and organic disorders, decreased libido, premature ejaculation, and erectile dysfunction can be seen in infertile male patients [182]., Somatosensory amplification and depression scores were found to be significantly higher in the group consisting of males who have difficulties in erectile function. Duration of infertility was correlated positively with somatosensory amplification and depression and negatively with erectile function scores [183]. In another study, the detection rate of depression in women was found to be higher than in men who are registered to IVF/ICSI program [184]. According to Algan [185], infertile women are more likely to show some psychological symptoms (such as somatization, obsessive-compulsive symptoms, interpersonal sensitivity, depression, anxiety, paranoid thought, psychoticism level, and general symptom level) than fertile women. However, Benli [186] did not find any difference between infertile and fertile women in terms of depression and anxiety. In infertile women, those who had negative feelings about the treatment process and those who thought that infertility had a negative impact on their life were found to have high scores of depression and anxiety, and those who did not have a source of support during the treatment process had high depression scores [186].

Another inner experience considered in terms of the consequences of infertility is hopelessness. There was no statistically significant difference between the hopelessness scores by gender [187,188]. In women, there was a positive relationship between age, duration of the marriage, the period of wanting a child, and hopelessness; while it was found only between the period of wanting a child and hopelessness in males [188]. In the process of infertility treatment, infertile women experience hopelessness as a result of negative treatment experience and helplessness by thinking that they will not be successful in treatment and in their life [189].

Furthermore, infertility may also lead to low self-esteem [149,179,190]. According to Dyer et al. [190], fertility determines the presence or absence of a woman, and therefore a woman might feel that she is “nothing” if she cannot give birth to a child. However, this seems not to apply only to women, because infertile couples as a whole have been found to have inferiority complexes [174].

Finally, childlessness may be related to body image. For instance, involuntarily childless women may be disappointed with their body, since they see it in relation to pregnancy and medical treatments and “the baby making machine” is just a target of treatments [83,86,87]. The study participants use descriptions such as “leprous”, “rotten”, “in vain” and others, when talking about their body [179]. Based on the matching of being a fertile woman, women stated that there were unpleasant changes in their bodies such as “psychically falling down”, “pilosity”, “hair loss”, and “deterioration of the body” during the period when they were the subject of infertility treatment as a remedy [64]. In contrast, voluntarily childless women may also be disappointed with their bodies, which is why some may use sterilization in a way to “fix” it [23,88].

All this being said, as with any life challenge, childlessness has the potential of strengthening one’s identity, leading to emotional growth [94,113]. As a survival mechanism, infertile women often use spiritual coping, adopt hiding mechanisms, resort to social/individual isolation, and hope to cope with the stress and psychosocial problems caused by infertility [125]. As a general finding, the need for psychological/psychotherapeutic or counseling services is high and it is very much recommended [191]. In addition, especially men may be left without support, for instance, during infertility treatments [92].

##### The Impact on Couple Relationships

Childlessness may bring challenges to couples’ relationships [92,95,176,192], for instance, by negatively impacting a couples’ intimate life [174,193]. Infertile people often have to reconcile their body and life according to many cycles of tests and treatments prescribed by doctors, allow a thorough examination of their sex life and genitals, listen to instructions on when to make love and when not, when and how to masturbate to inject sperm into a plastic container, or if they want to adopt a child, allow the authorities to assess their suitability to be parents [194]. Voluntarily childless people, in turn, may find it challenging to find a partner who shares their choice regarding not having children [126]. Accordingly, voluntary childlessness impacts who one chooses as his/her partner [88].

The initial reaction to involuntary infertility sometimes could lead to a cooling of the relationship, rejection of the partner, even a proposal to divorce [149,195]. Similarly, childlessness in Turkey can lead to divorce or a fellow/second spouse. Although monogamy is legally valid in Turkey, in the case of childlessness, provided that only one of the women is legally married to her husband, these two women may have to live together. The other spouse, who is not legally married to her husband, is usually only religiously married. Therefore, the demand for infertility treatments is very high, especially in lower socioeconomic environments, Eastern and Southeastern Anatolia regions, to avoid divorce and not to live with a second/fellow wife. On the contrary, in some cases, infertility might also strengthen the relationship between a couple, bringing spiritual closeness [92,176,192,195].

Most of the studies focus on comparing outcomes for the adjustment of couples with and without children. Living in the city, having a profession, having a good income, and having more years of marriage are predictors of marital adjustment in primary infertile women [196]. There was no significant difference between infertility and control groups in terms of sexual function. Still, women and men in the infertility group had more problems in terms of dyadic adjustment than the control group [197]. In another study, according to the variable of having children, there was no difference between couples in terms of marital adjustment and dyadic adjustment, as well as sexual satisfaction in women and men did not differ based on this variable [198]. On the contrary, another study showed that women affected by infertility have worse dyadic adjustment than men [199]. Although women in the infertility group reported a higher decrease in marital adjustment than infertile men, this was not different from the control group [200]. In another study, there was no difference between families with children and childless families in terms of depression, but marital satisfaction was higher in childless couples [201].

According to a research study conducted by Tarlatzis et al. [191] among infertile couples in Greece, women showed a high defensive anxiety, had psychosomatic symptoms, suffered from previous abortion guilt and they seemed to have more challenges in social adjustment processes. Both spouses were discovered to be emotionally disturbed, although not severely, or had psychological problems, seemed to have special needs and fears, and they reported sexual dysfunction (50% of the cases) mostly associated with a degree of deterioration in their marriage. Finally, the couples coming from rural areas seemed to be more severely burdened by the traditional rules.

Mental health professionals and psychotherapists together with educators will greatly benefit from becoming aware of such background forces at play. This will enable them to better support clients so that they can move from seeing each other as the means to get out of their inner conflicts and meet each other afresh healing PTSD issues that may be revealed in their past.

##### The Impact on Social Life

Society still perceives infertility stereotypically, so the infertile family is prone to hide its problems and to maintain distance from society [124]. Living without children is incomplete and the family feels socially excluded [194]. Families often avoid meetings with relatives and friends or co-workers. Due to the negative public attitude, infertile couples hide this problem for fear of social isolation [174]. The voluntary childlessness chosen by women is often compared with immorality; women are compared with cuckoos who are selfish who throw their competitors out of the nest when they are born and tend to lay eggs in the nests of other species and not raise their children [149].

A significant proportion of women facing infertility suffer from stigmatization and insults [193,202], and sometimes from physical violence [203]. Accordingly, childlessness forms gender inequality because, as mentioned above, women are usually judged for not having children, perceived as selfish, or judged for their inability to conceive; however, childless men are seen as interesting, and they are not stigmatized [85,149]. Furthermore, during IVF, women also go through more in the process, not only physically but mentally [204,205,206].

Voluntary and involuntary childlessness may, thus, impact one’s relationship with the broader social network, such as friends and colleagues [61,82,86,88,192]. Studies show that constant questions and suggestions from the social environment present one of the most uncomfortable situations for childless couples [207]. In addition to these, pity is also felt for childless women [208]. Involuntarily childless people may end up isolating themselves, for instance, because they feel alone with their experience [61,94,174,177,203]. Voluntarily childless people, in turn, may feel they need to “live in a closet” with their choice due to the critique that they receive from their social network [82,85,209].

All the above-mentioned aspects emphasize the need for psychosocial support and many studies reveal the importance of receiving support from either one’s peers, social network, health professionals, or others, both for childless women and men [86,95,176,191,192,209,210,211,212]. Even working as a volunteer (related to childlessness) or receiving peer support online (e.g., peer support group, blogging about childlessness) may help to maintain and strengthen one’s hope in the midst of childlessness [178,211,212]. However, women and men may have different coping strategies [192], and some men may benefit more from concrete support (e.g., information) than from emotional support [92]. Finally, some people may, unfortunately, face challenges with receiving social support, which leaves them coping with childlessness unaided [169].

In one Turkish study aimed to investigate the relation of anxiety and depressive symptoms with perceived social support according to gender within infertile couples, the depressive symptom level of the sample was not indicative of clinical depression, and their anxiety was within normal limits. It was observed that female participants were more anxious than males and that as the perceived social support of the couples increased, anxiety and depressive symptoms decreased. In terms of perceived social support, it was determined that the special support, friend support, and total social support scores of the infertility group originating from both genders were higher than the female-originated infertility group. It was determined that the specific support perceived by the male-originated infertility group was higher than the female-originated infertility group [213]. In another study, it was determined that primary infertile women have a high level of perceived social support from their friends and spouses, and especially their families, that the level of psychologically negative effects from infertility is moderate, and that as the perceived social support of women increases, the level of negative infertility decreases significantly [214].

Finally, the whole society can become the members who will connect with each other openly based on trust and mutual benefit. We can all become vehicles of health and not agents of viral angst.

##### Seeking Solutions and Adaptation

Having already seen the unpleasant consequences of being childless, people have always resorted to alternative solutions in their efforts to relieve their stress or adapt. The first stage of adaptation begins when they learn the news about infertility, especially when involuntary. They start to probe into the causes and try to learn as much as they can. As a result, it is seen that infertile women have more medical/biological knowledge about fertility issues than fertile women [215]. In addition to raising awareness, there are also other aspects in the adaptation process. In the early stages, they try mild ways of treatment. For example, in Turkey, women use traditional methods (e.g., royal jelly, pills made from herbs and spices, placing balls of herbs in the uterus, straightening the uterus by hand) not recognized by modern medicine and sometimes at the expense of their health before [216] and during the fertility treatment [217]. Similarly, it has been found that most infertile women treated at IVF centers apply complementary or supportive care practices and do not tell their doctors about these practices, such as eating a herbal mixture or drinking the juice of a herbal mixture [218]. Another study found that women attending the infertility outpatient clinic use traditional methods to facilitate pregnancy. While the most commonly used traditional methods are Hodja/Shiha blowing and waist-tying, most spouses prefer to eat a mixture of honey [219]. Women with low socioeconomic status have higher levels of using traditional methods to facilitate pregnancy [219]. Although access to modern methods in the treatment of infertility has become quite easy, traditional methods can still be used in eastern Turkey [220].

A growing majority of infertile women resort to assisted technology (i.e., IVF, IUI, and ICSI) to “treat” the problem. It has been determined that women experience difficulties at every stage of the treatment process and difficulties in their family, social environment, and work due to the side-effects of the treatment process [221]. For example, it was concluded that IVF treatment caused more anxiety and somatization and affects quality of life more negatively than IUI treatment [185]. Interestingly, lower- and lower-middle-class women, who were hospitalized for infertility treatment, expressed gratitude to their doctors. They were freed from heavy housework, abuse, and boredom by being with other women who share similar experiences. In Turkey, where the expected housework from women is high, in-patient infertility treatment provides benefits as a part of personal care [116].

When all such efforts do not come to fruition, childless couples seek alternative ways of being a parent, such as surrogacy and adoption. After suffering from involuntary childlessness, adopting a child may make life valuable and happy again [113,222]. However, attitudes towards adoption differ in different countries. In a study evaluating the views of infertile couples on becoming parents conducted in Turkey, it was seen that most of the couples had an opinion that they would continue treatment until they had their own children [223]. Other views were determined as living a life without having children and adopting a baby/child [223].

Besides adoption, raising the children of relatives can also be part of the adaptation. Working with children or raising the children of relatives in older women can be a compensation for not having their own children [84,160]. Being such “a substitute mother”, “a social mother”, or “a godmother” can be important for the childless woman as well as the child [84].

According to Lechner et al. [210], there is a significant difference between those who resort to passive adaptation solutions and those who take a more active role. Regression (psycho)analysis has shown the concepts of passive coping style and dissatisfaction with social support were positively associated with health complaints, depression, anxiety, and complicated grief. The concept of an active coping style was negatively associated with depression, anxiety, and complicated grief. In conclusion, we would say that psychosocial interventions should be continued after the “childlessness reality” has become definite. By teaching couples how to cope actively with their childlessness and how to ask for support, the negative consequences may be decreased. What is more, the healing process involved could also offer added value to the quality of life of the childless people as it will relieve the tension of the hidden causes which may affect other life/health aspects.

This is also an invitation for all of us to turn to salutogenic models which empower individuals and societies, cultivate resilience, and discover meaning in all acts of life experiences so that we can release the inner potentiality inherent in all of us. Attitudinal change programs can be designed and tested to work with different populations in this direction of healthy adaptation that leads to consciousness evolution.

## 4. Final Thoughts

The study of childlessness as a concept is the base that allows a deep understanding of the background forces which explain behaviors, mindsets, practices, and challenges of today and can also lead to solutions to eons-long phenomena. As we have seen from a linguistic analysis of the concept, childlessness carries quite a negative connotation that evokes condemnation as well as sympathy from others. Contrarily, parenthood and all related antonyms have a positive meaning and are associated with respect, richness, and fulfillment. The responsibility for childlessness from older times still lingers more on the women’s side, so we can assume that women are the ones who also feel more pressure related to the consequences of being childless. However, consequences of childlessness are more studied in women’s samples in all analyzed countries.

The concept itself was influenced by existing social norms and religious doctrines and is slowly changing because of the changes in the social norms and gender-related movements. We could find many myths, epics, and tales in folklore about how people tried to escape childlessness by following rituals and superstitions or trying to foretell the gender of their baby. Some of the rituals are still practiced at least in some parts of Turkey. The image of parenthood is fully supported in the media and childless people are often stigmatized. However, contraception is presented from a more positive perspective than before.

Having children and having boys in some regions in Greece is still seen as a continuation of survival of the nation and as a continuation of one’s existence. Childless individuals are assessed as egoistic, sinners, and enemies of the nation, with a slight exception in Finland. All supportive childbearing solutions and adoption are legal in analyzed countries with the exception of surrogacy, which is only legal in Greece. Nonetheless, the reproduction rates in all analyzed countries are gradually decreasing.

Despite the causes of childlessness, whether it is involuntary, voluntary, or determined by circumstances, it brings negative consequences for a childless individual. Childlessness negatively affects psychological well-being, a couple’s relationship, and social life. Moreover, it challenges individuals to seek solutions and adapt to the reality of being childless.

Consistent with the purpose of this concept analysis is to explore childlessness and provide understanding to professionals involved in the field of infertility. It could be stated that previous research has offered various perspectives on the nature of childlessness and it can be seen as a developmental or traumatic crisis that can have a severe impact on one’s mental health [60,61]. However, today, childlessness is seen as “a life course process across a series of decision and bifurcation points” [73].

### Limitations and Future Directions

This concept analysis was based on the study of childlessness in Greek, Turkish, Finnish, Lithuanian, and Maltese languages and cultures. Although it is full of meaning, it is incomplete as it would be necessary to explore the depths of the concept in other languages, especially the ones spoken in non-European countries and those that have different religions and developed different civilizations. This would allow a greater richness due to the multi/transcultural element. Furthermore, more research needs to be conducted in the field of male childlessness, as this kind of research is still often missing. In addition, while studies on male infertility are mostly carried out from a medical point of view, studies on female infertility are carried out from a psychological point of view. For this reason, we can say that it would be beneficial to ensure gender equality in terms of the fields of study. Further research into the two opposite philosophical positions but also socio/political movements of pronatalism and antinatalism remains to be carried out.

## 5. Conclusions

Understanding the concept of childlessness allows theoreticians and academics to ask new questions such as the questions of to what extent is childbearing a voluntary or involuntary decision, which will present the long-standing philosophical but also psychological debates of free will or conscious and unconscious or collective unconscious aspects. Besides, the theme of decision making is a most interesting issue to explore especially when linked to having a child or not. Are we actually free to make wise decisions? Do we really have a choice? Finally, do we actually welcome our children or do we still “use” them as a survival strategy to satisfy our unmet needs or appease our individual or collective fear of rejection or marginalization and stigmatization? Researchers may be inspired to develop tools or come up with therapeutic practices, which will be able to provide good answers to such challenges where today we see intervention practices based on the long-standing traumas of prejudice. Professionals studying the concept can better understand the angst experienced by people who come to their practice and might be better prepared to offer them optimum support, while educators, social reformers, and society leaders can design and implement projects that could release the trapped energy and allow better relations among humans, within the family, and a better understanding of the human–nature connection for life sustainability, promoting health for the generations living now and those in the future.

## Data Availability

The datasets analyzed during the current study are available from the corresponding author upon reasonable request.
